# Cytoprotective Effect of Ascorbic Acid and Rutin against Oxidative Changes in the Proteome of Skin Fibroblasts Cultured in a Three-Dimensional System

**DOI:** 10.3390/nu12041074

**Published:** 2020-04-13

**Authors:** Agnieszka Gęgotek, Iwona Jarocka-Karpowicz, Elżbieta Skrzydlewska

**Affiliations:** Department of Analytical Chemistry, Medical University of Bialystok, Mickiewicza 2d, 15-222 Bialystok, Poland; iwona.jarocka-karpowicz@umb.edu.pl (I.J.-K.); elzbieta.skrzydlewska@umb.edu.pl (E.S.)

**Keywords:** ascorbic acid, proteome, rutin, skin fibroblasts, three-dimensional culture, UV radiation

## Abstract

The combination of ascorbic acid and rutin, commonly used in oral preparations for their antioxidant and anti-inflammatory properties, can also be used to protect skin cells from the effects of UV radiation in sunlight. Here, we tested the potential protective effect of ascorbic acid and rutin used together in UVB-irradiated human skin fibroblasts, and assessed the proteomic profile of these cells, grown in a three-dimensional (3D) system. Proteomic findings revealed a combined effect of ascorbic acid and rutin in UV-irradiated fibroblasts against overexpression of pro-inflammatory signaling proteins and DNA reorganization/expression. These effects were not observed when cells were treated with either compounds alone. The antioxidant effects of ascorbic acid and rutin also prevented protein modifications by lipid peroxidation products. Further, ascorbic acid stimulated rutin-protein adduct formation, which supports intra/extracellular signaling and the Nrf2/ARE antioxidant pathway, contributing to the protective effects against UV-induced oxidative stress. The combined effect of ascorbic acid and rutin suggests that this combination of compounds is potentially effective against skin damage caused by UV radiation.

## 1. Introduction

UV radiation contained in sunlight is one of the common harmful environmental factors to which our skin is exposed daily. As a result, this leads to the disruption of many metabolic pathways in cells responsible for building skin layers, which has been widely documented in the literature [1,2]. UV radiation, which mostly reaches the skin, contains high-energy UVB (280–320 nm) and highly penetrating UVA (320–400 nm). Both types of radiations lead to different biological effects; however, they have a common feature of intensifying the generation of the reactive oxygen species (ROS) [3]. As a result, irreversible oxidative changes in intracellular molecular structures might be observed, as is seen in the case of nucleic acids, which causes DNA mutations and lead to the development of skin cancers [4,5]. At the same time, UV-induced ROS production has a destructive effect on skin cells by being able to induce the peroxidation of proteins and lipids, which directly leads to disruption of the structure of biological membranes and the formation of secondary signal transduction molecules, such as 4-hydroxyalkenals [2]. In addition, intracellular redox imbalance leads to the modification of protein structures [6]. Oxidative modifications of proteins affect their conformation, causing changes in their activity, which is particularly important in the case of proteins with antioxidant and DNA repair properties and also in signal molecules that are involved in the pro-inflammatory and proapoptotic response of skin cells [7,8].

Fibroblasts are one of the primary cell types involved in building human skin, but their localization to the inner layer of the skin, the dermis, makes them partially protected from external factors by epidermal cells. Due to this protection, fibroblasts are more sensitive to the UV-induced changes than epidermal keratinocytes [9]. Therefore, fibroblasts are characterized by well-developed cytoprotective system aimed at intracellular homeostasis maintenance. The most important components of this system are enzymatic and non-enzymatic antioxidants, redox sensitive transcriptional factors, such as Nrf2 or Ref1, which are activated by signal transduction pathways, including mitogen-activated protein (MAP)-dependent signaling kinases (e.g., ERK1/2) [10]. Additional mechanisms of skin fibroblast cytoprotection is the ability of these cells to form multilayer construction and communication between cells by signal factor transmission [11,12]. However, due to the high risk of UV exposure, natural cell defense systems are insufficient, causing a constant need for the daily use of skin protection compounds that are able to support the protective mechanisms. Examples of such substances include natural plant antioxidants, such as often used in pharmaceutic formulas—rutin and common nutrient ascorbic acid. Ascorbic acid, known as vitamin C, has been identified as an effective skin protector against sunburn induced inflammation [13,14]. In addition, ascorbic acid normalizes the polarization of the mitochondrial membrane and ensures proper mitochondrial respiration in these organelles when fibroblasts are exposed to UV-irradiation [15]. Ascorbic acid is also necessary for the biosynthesis of collagen in fibroblasts and the formation of well-organized multilayer structuring of the dermis [16], which has been shown in the case of keratinocytes grown in 3D [17,18]. On the other hand, rutin, as a polyphenol, effects cellular metabolism not only via reactive oxygen species scavenging, but also by prooxidant enzymes inactivation [19]. Moreover, its antioxidant action is also based on the activation of cytoprotective transcription factor Nrf2 [19]. It has been shown that rutin prevents UV-induced changes in skin cell membrane function and, by inhibiting the activity of cyclooxygenases and lipoxygenases as well as non-enzymatic lipid mediators generation, inhibits pro-inflammatory signaling [20,21]. Moreover, rutin exerts cytoprotective effects on cells exposed to various types of physical factors by substantially increasing their viability [22,23]. Additionally, rutin exerts cytoprotective effects on cells exposed to different types of radiation by substantially increasing their viability [16]. Literature data show that the range of toxic concentration of rutin depends on the type of cells, and it has been indicated that rutin has a toxic effect in the range of 125–250 μM in the case of cancer cells [24,25] and, with respect to keratinocytes (HaCaT), toxic effects of rutin are achieved at concentrations even above 1 mM [26].

To properly describe the changes in biological mechanisms that occur when living organisms are treated with chemical substances that have potentially protective properties under various stress conditions, it is important to choose the right model for the experiment. As was shown in previous studies, usage of an in vitro model based on a two-dimensional (2D) culture rather than a three-dimensional (3D) culture for skin cells, such as fibroblasts, is much less reflective of the processes occurring in the skin tissue. Specifically, the cellular metabolism, inter-cellular communication, shape and mobility are not as well represented in 2D cultures as they are in 3D models [27,28,29]. The use of a 3D culture model is especially meaningful in the case of skin cells exposed to UV radiation, a scenario in which the mechanism of action is dependent on multilayer structure penetration [1,30]. Alternatively, the interaction of ascorbic acid with rutin has primarily been demonstrated in terms of anti-inflammatory and vascular sealing effects when administered orally [31]. However, emerging data on the in vitro treatment of skin cells with ascorbic acid and rutin suggest that such a mixture is a potentially beneficial topical protection against skin damage caused by UV radiation [30,32]. Therefore, the aim of this study was to examine the cooperation of ascorbic acid and rutin in the protection of the proteomic profile in UVB-irradiated human skin fibroblasts cultured in a 3D system.

## 2. Material and Methods

### 2.1. Fibroblast Treatment

Fibroblasts line CRL-1474 isolated from human skin was obtained from the American Type Culture Collection (ATCC). Cells were cultured in a two-dimensional model in a humidified atmosphere of 5% CO_2_ at 37 °C in a medium composed of Dulbecco’s Modified Eagle Medium (DMEM) with 10% fetal bovine serum (FBS) and supplemented with 50 μg/mL streptomycin and 50 U/mL penicillin. Sterile reagents were obtained from Gibco (Grand Island, NY). When the cells reached 9 passages and 90% confluence, they were seeded in 24-wells plates (5 × 10^5^ cells/well) with AlgiMatrix gel (Life Technologies, Carlsbad, CA, USA) to create a three-dimensional model. Following a four day incubation, cells were exposed to UVB (312 nm) radiation in a total dose of 200 mJ/cm^2^ (Bio-Link Crosslinker BLX 312, Vilber Lourmat, Germany). To analyze the effect of ascorbic acid and rutin on UV radiation, cells were incubated 24 h in a medium containing 100 µM ascorbic acid and/or 25 µM rutin in 0.1% dimethyl sulfoxide (DMSO). Control cells were cultured in parallel with no treatment. To control the effects of the studied factors on cells viability, cell metabolic activity was measured using an MTT test [33].

Following incubation, fibroblasts were collected from 3D gel with AlgiMatrix™ dissolving buffer (Life Technologies, Carlsbad, CA, USA), lysed by sonication on ice, and centrifuged (15 min, 12,000× *g*). The total protein content in lysates was measured using a Bradford assay [34].

### 2.2. Protein Separation and Analysis

Lysates were denatured by mixing with a Laemmli buffer supplemented with 5% 2-mercaptoethanol in a 1:1 ratio and heating at 95 °C for 10 min. Samples were then separated on 10% Tris-Glycine SDS-PAGE gels and stained overnight with Coomassie Brilliant Blue R-250. Complete lanes were cut out of the gel, sliced into 8 sections, and in-gel digested overnight with trypsin (Promega, Madison, WI, USA). The obtained peptide mixture was extracted from the gel and dissolved in 5% ACN + 0.1% formic acid (FA). Ultimate 3000 (Dionex, Idstein, Germany) with a 150 mm x 75 µm PepMap RSLC capillary analytical C18 column (Dionex, LC Packings) was used to separate peptides that were analyzed using a QExactive HF mass spectrometer with an electrospray ionization source (ESI) (Thermo Fisher Scientific, Bremen, Germany). The mass spectrometer was externally calibrated and operated in positive and data-dependent modes. Survey MS scans were conducted in the 200–2000 *m*/*z* range with a resolution of 120,000. In subsequent scans, the top ten most intense ions were isolated, fragmented, and analyzed at 30,000 resolution. A 10 s dynamic exclusion window was applied, and an isolation window of 4 *m*/*z* and one microscan was used to collect suitable tandem mass spectra.

### 2.3. Protein Identification and Label-Free Quantification

Raw data generated from the liquid chromatography-mass spectrometry (LC-MS/MS) analysis were processed using Proteome Discoverer 2.0 (Thermo Fisher Scientific, Bremen, Germany), and input data were searched against the UniProtKB-SwissProt database (taxonomy: Homo sapiens, release 2018-04). Parameters of peptide mass tolerance set to 10 ppm, MS/MS mass tolerance set to 0.02 Da, and up to two allowed missed cleavages were used for protein identification. Protein quantification was performed using the peak area analysis. Cysteine carbamidomethylation/carboxymethylation and methionine oxidation were set as dynamic modifications. Only proteins with at least two unique peptides identified were taken for further analysis. The formation of rutin or lipid peroxidation products adducts with protein was detected as changes in the mass of the individual amino acid residues as follows: rutin—cysteine mass increase by 610.153, MDA—lysine mass increase by 72.021, 4-ONE—cysteine mass increase by 154.206, and 4-HNE—cysteine/lysine/histidine mass increase by 156.115 [35,36]. The number of peptides containing each modification was considered as the amount of each type of adducts and was presented in relation to the values found in the control cells.

### 2.4. Statistical Analysis

Analyses of each sample were performed in three independent experiments. Results from individual protein label-free quantification were normalized by the sample sum, log transformed, and analyzed using the standard statistical analysis methods, including T-test, principal component analysis (PCA), and heatmap creation using MetaboAnalyst 4.0 software (http://www.metaboanalyst.ca) [37]. The top 50 proteins used for heatmap creation were selected according to the lowest *p*-value computed during analysis of variance (ANOVA). Biological and molecular functions of proteins were identified using the Panther Classification System (http://pantherdb.org) [38], and complex analyses were conducted using ComplexBrowser (http://computproteomics.bmb.sdu.dk/Apps/ComplexBrowser/) [39].

## 3. Results

**Figure 1:**
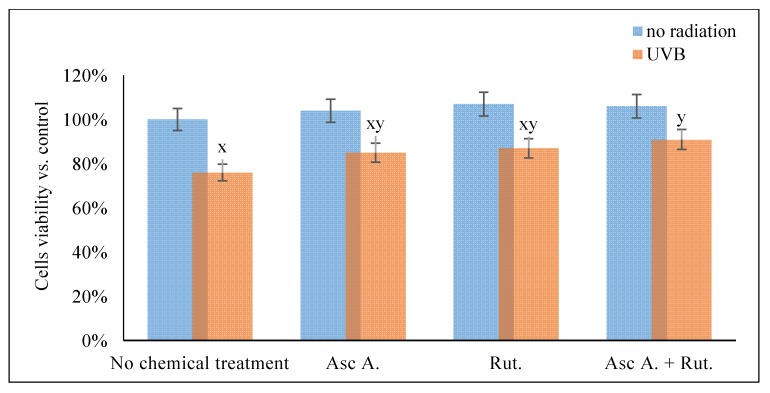
The viability of the 3D cultured fibroblasts exposed to UVB irradiation (200 mJ/cm^2^) and treated with ascorbic acid [100 µM] and/or rutin [25 µM]. Abbreviations: Asc, ascorbic acid; Rut, rutin; Ctr, control. Mean values ± SD of three independent experiments are presented. x, statistically significant differences vs. non-irradiated group without chemical treatment, *p* < 0.05; y, statistically significant differences vs. non-irradiated group, *p* < 0.05.

The results obtained in this study showed that the viability of 3D cultured cells measured by MTT test was significantly decreased by UVB radiation (76% compared to control cells), while ascorbic acid and/or rutin treatment partially prevent these changes, observed as a 85% and 87% viability, respectively, for ascorbic acid and rutin treated cells, and 91% in cells treated with rutin and ascorbic acid together (Figure 1).

The date results presented below from the analysis of 899 identified proteins (Supplementary Table S1) showed that ascorbic acid and rutin treatment caused significant changes in the proteomic profile of fibroblasts exposed to UVB radiation. These changes occurred at the protein level, and contributed to visible adducts formation within lipid peroxidation products in the protein structure.

**Figure 2:**
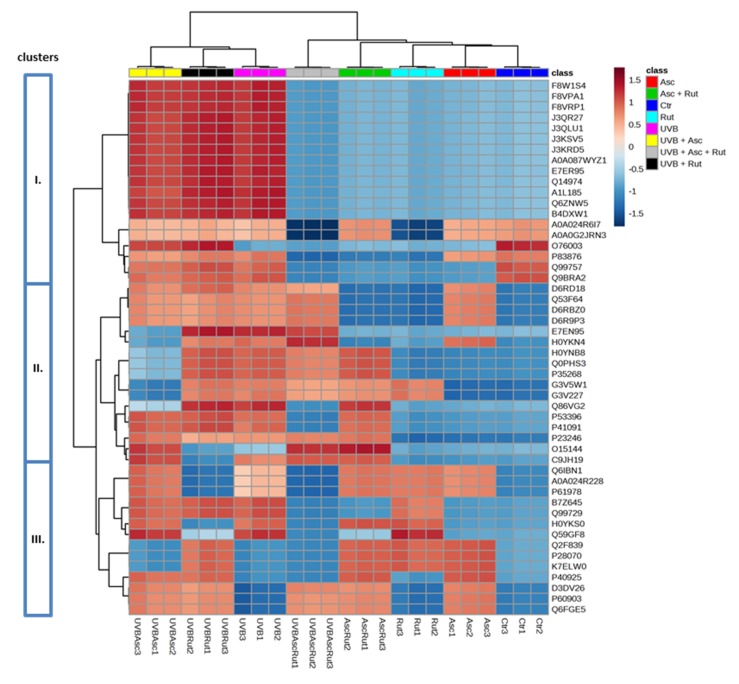
Heat map and clustering for the top 50 proteins from the 3D cultured fibroblasts exposed to UVB irradiation (200 mJ/cm^2^) and treated with ascorbic acid [100 µM] and/or rutin [25 µM]. Protein expression levels (log transformed) were scaled to the row sum. Abbreviations: Asc, ascorbic acid; Rut, rutin; Ctr, control. Analysis made using MetaboAnalyst 4.0.

The ascorbic acid and rutin induced changes in the protein expression of UV-irradiated fibroblasts were hierarchically clustered, as is shown for the top 50 proteins (Figure 2). Therefore, it was possible to distinguish 3 larger clusters containing:

- Isoforms of cytosolic dipeptidase (J3QR27, J3QLU1, J3KSV5, J3KRD5, A0A087WYZ1), citrate synthase (F8W1S4, F8VPA1, F8VRP1), GDP-D-glucose phosphorylase (A1L185, Q6ZNW5), sepin (A0A024R6I7, A0A0G2JRN3), UDP-glucose 6-dehydrogenase (E7ER95), importin (Q14974), actin-related protein 3 (B4DXW1), thioredoxin (P83876, Q99757, Q9BRA2), and glutaredoxin (O76003);
- Isoforms of nuclear ribonucleoprotein A/B (D6RD18, Q53F64, D6RBZ0, D6R9P3), annexin (H0YNB8, H0YKN4), filamin (E7EN95), glucose-6-phosphate dehydrogenase (Q0PHS3), ATP-citrate synthase (P53396), proline/glutamine-rich splicing factor (Q86VG2, P23246), cathepsin D (C9JH19), actin-related protein 2 (O15144), 60S ribosomal protein (P35268), tryptophanyl-tRNA synthetase (G3V5W1, G3V227), and translation initiation factor 2 (P41091);
- Isoforms of nuclear ribonucleoprotein A/B, K, Q (Q99729, Q6IBN1, A0A024R228, P61978, B7Z645), protein/nucleic acid deglycase (K7ELW0), malate dehydrogenase (P40925), proteasome endopeptidase complex (H0YKS0, P28070), heat shock protein 70 (Q2F839, Q59GF8), and calcium binding protein S100 (D3DV26, P60903, Q6FGE5).

**Figure 3:**
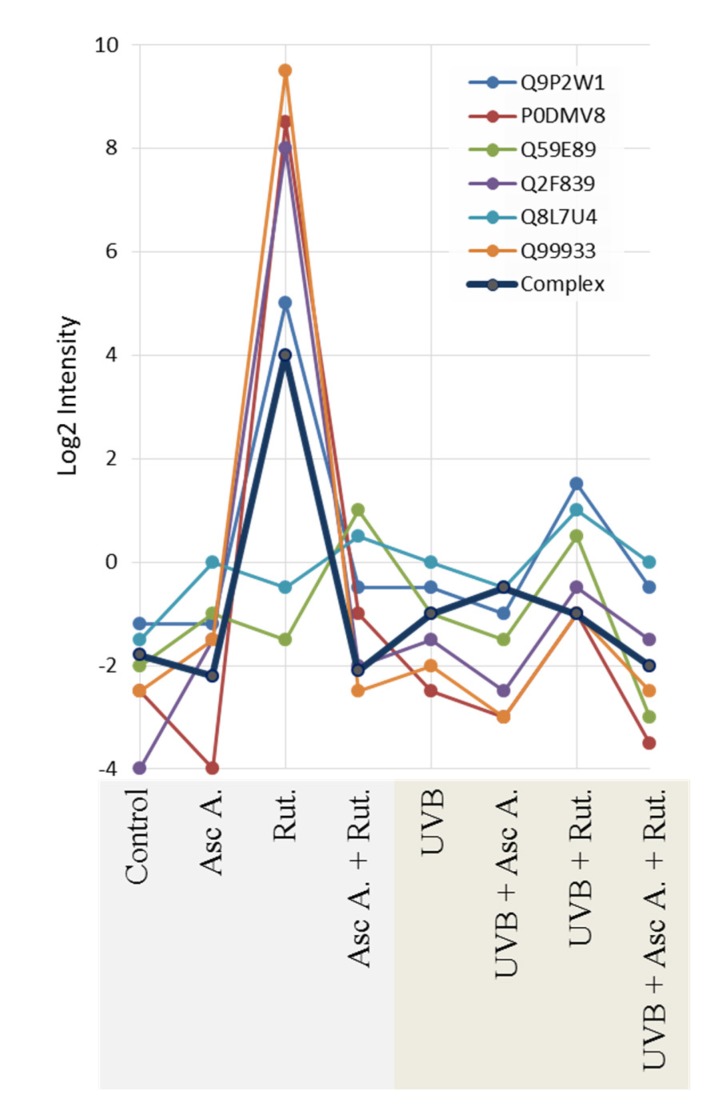
The intensity of the proteins creating complex with active heat shock protein 90 (HSP90) in 3D cultured fibroblasts treated with rutin [25 µM] and/or ascorbic acid [100 µM], and exposed to UVB irradiation (200 mJ/cm^2^). Abbreviations: Asc A, ascorbic acid; Rut, rutin. Proteins: P0DMV8—heat shock protein 70 (HSP70); Q2F839—heat shock protein 90 (HSP90); Q59E89—heat shock protein 40 (HSP40); Q8L7U4—co-chaperone protein p23; Q99933—BAG family molecular chaperone regulator 1; Q9P2W1—homologous-pairing protein 2 (HOP2). Analysis made using ComplexBrowser.

**Figure 4:**
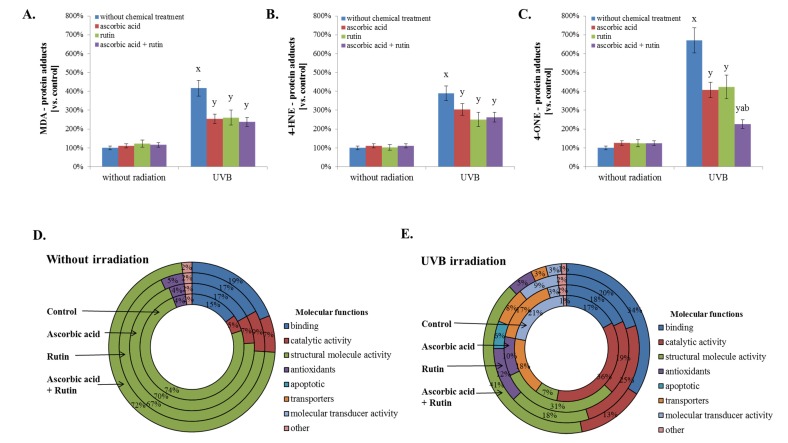
The level of protein adducts with selected lipid peroxidation products, such as malondialdehyde (MDA) (**A**), 4-hydroxynonenal (4-HNE) (**B**), and 4-oxynonenal (4-ONE) (**C**); the molecular functions of these proteins in 3D cultured fibroblasts were treated with rutin [25 µM] and/or ascorbic acid [100 µM] (**D**), and exposed to UVB irradiation (200 mJ/cm^2^) (**E**). Analysis made based on data from the Panther Classification System. Mean values ± SD of three independent experiments are presented. ^x^ statistically significant differences vs. non-treated group, *p* < 0.05; ^y^ statistically significant differences vs. group without chemical treatment, *p* < 0.05; ^a^ statistically significant differences vs. ascorbic acid treated group, *p* < 0.05; ^b^ statistically significant differences vs. rutin treated group, *p* < 0.05.

Moreover, UV radiation, as well as ascorbic acid and/or rutin treatment, affects the activity of proteins, creating active complexes with heat shock protein 90 (HSP90, Q2F839). These impacted proteins include the aforementioned HSP70 (P0DMV8), HSP40 (HSP40, Q59E89), co-chaperone protein p23 (Q8L7U4), BAG family molecular chaperone regulator 1 (Q99933), and homologous-pairing protein 2 (HOP2, Q9P2W1). Rutin was identified as the primary factor that enhances the HSP90 complex; however, this effect was not observed in UVB-irradiated fibroblasts (Figure 3). As a result of UVB irradiation, a strong (up to 7-fold) increase in lipid peroxidation protein adduct product levels was observed (Figure 4). However, the treatment of cells with ascorbic acid and rutin significantly reduced these protein modifications. In addition, the use of a mixture of ascorbic acid and rutin prevented the formation of 4-ONE-protein adducts to a greater extent than when these substances were used alone. Further, the described protein modification was observed on molecules with varying functions. While UVB caused the modification of proteins with catalytic and molecular transducer activity and transporters, the ascorbic acid and rutin mixture shifted these changes towards proteins with structural molecule activity, which was unique when compared to results obtained for each compound used separately (Figure 4).

**Figure 5:**
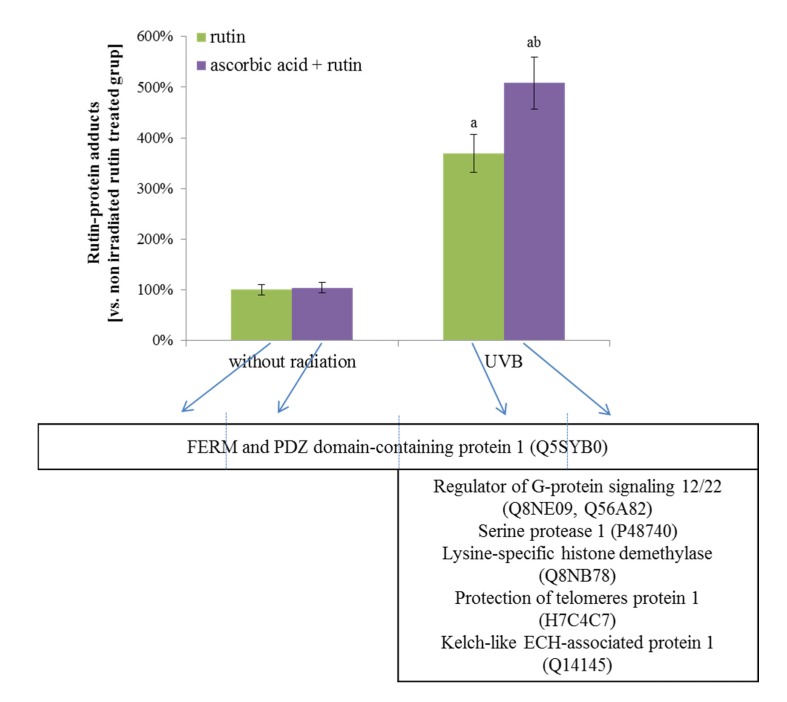
The level of rutin-protein adducts and the names of these proteins in 3D cultured fibroblasts treated with rutin [25 µM] and/or ascorbic acid [100 µM], and exposed to UVB irradiation (200 mJ/cm^2^). Analyses were made based on label free quantification. Mean values ± SD of three independent experiments are presented. ^a^ statistically significant differences vs. non-irradiated group, *p* < 0.05; ^b^ statistically significant differences vs. rutin treated group exposed to the UVB irradiation, *p* < 0.05.

Another type of protein modification observed after the treatment of fibroblasts with rutin, ascorbic acid, and physical factors was the creation of adducts with rutin. UVB radiation significantly increased the level of these protein modifications, but the highest levels of rutin-protein adduct formation were observed in UV-irradiated fibroblasts treated with rutin and ascorbic acid together (Figure 3). There were also the differences in the types of proteins that created adducts with rutin. The main proteins modified by rutin were FERM and the PDZ domain-containing protein 1 (Q5SYB0), which was found in all samples. The following UVB radiation proteins, such as the regulator of G-protein signaling 12/22 (RGS12 and RGS22, Q56A82 and Q8NE09), serine protease 1 (P48740), lysine-specific histone demethylase (Q8NB78), protection of telomere protein 1 (H7C4C7) and cytosolic inhibitor Nrf2–Keap1 protein (Kelch-like protein 1 ECH, Q14145), were also modified by rutin regardless of ascorbic acid supplementation (Figure 5).

## 4. Discussion

The harmful effect of UV irradiation on skin cells can be reduced via application of effective cytoprotective compounds. Therefore, in this study, the cooperation of two well-known antioxidants, ascorbic acid supplied with food and rutin that is often used in pharmacy, were assessed for their role in the protection of the proteome in UVB-irradiated human skin fibroblasts. The results of this study indicate significant changes in the proteomic profile of UVB-irradiated fibroblasts treated with ascorbic acid and rutin separately, as well as in combination. However, as shown in Figure 1, for the 50 top proteins, the use of ascorbic acid and rutin together leads to a similar proteomic profile as is seen in the control fibroblasts, which differentiate from the proteome of UVB-irradiated cells. This alteration in the proteomic profile is strongly visible in the case of proteins that suppress phospholipase A2, annexins (H0YNB8, H0YKN4), and thus becomes a natural defense of cells against UV-induced inflammation and prepares the skin tissue for fibrinolysis [40,41]. While UV radiation strongly enhanced annexin expression, ascorbic acid and rutin restored their levels to ones that are comparable to non-irradiated cells, suggesting a role in anti-inflammatory signaling and skin cell protection against inflammation.

Moreover, similar changes were observed for proteins that are connected with gene expression, protein biosynthesis, and cell development, including nuclear ribonucleoprotein A/B and 60S ribosomal protein. In the case of nuclear ribonucleoprotein A/B, which is involved in pre-mRNA processing, metabolism, and transport, the ascorbic acid and rutin decreased expression. This was also the case in studies where polyphenol (rutin or epigallocatechin-3-gallate) was used in UV-irradiated skin cells or cancer cells [42], suggesting an association of ribonucleoprotein A/B with arresting cell proliferation due to a lack of significant damage resulting from UV irradiation. Moreover, synergistic action of ascorbic acid and rutin leads to the overexpression of splicing regulators, such as the pre-mRNA splicing factors Q86VG2 and P23246, which are proline/glutamine-rich splicing factors required in early spliceosome formation [43]. This cell reaction was not observed when ascorbic acid or rutin were used separately, but the increase in the spliceosome formation has been previously found in the cases of other antioxidants/polyphenols [44]. Enhanced splicing following ascorbic acid and rutin treatment might be the cellular defense response, which leads to increased expression of protein isoforms, protecting them against oxidative modifications and loss of function, as has been observed in cancer cells during anticancer therapy [45].

Another group of proteins that are up regulated under stress conditions and down regulated following ascorbic acid and rutin combination treatment are proteins involved in energy metabolism. While UV radiation increases the level of enzymes taking part in glucose metabolism, ascorbic acid-rutin cooperation is stronger than either compound used separately to prevent these changes. In the case of glucose-6-phosphate dehydrogenase, an enzyme participating in the pentose phosphate pathway [46], that activity is inhibited by many known polyphenols [47]. Ascorbic acid-rutin synergy also inhibits this enzyme, significantly redirecting cellular metabolism to glycolysis and supporting energy management in cells. However, ascorbic acid-rutin cooperation prevents the action of GDP-D-glucose phosphorylase, an enzyme that is partially involved in the prevention of glucose incorporation in the place of mannose residues [48], and stops protein over-labeling. In the case of proteins coexisting in the organism, intracellular signaling via decreasing mannose-protein expression may indicate that combination ascorbic acid-rutin treatment induces prevention of inflammatory response.

Also, the protein-folding processes coordinated by heat shock proteins 70 and 90 (HSP70; HSP90) are protected from UV-induced disturbances by ascorbic acid and rutin. UV radiation, causing disruption in protein structures, leads to an increase in protein chaperone (HSP70, HSP32, HSP90) level and activity [36,49]. HSP70 interacts with all proteins in their unfolded, misfolded, or aggregated states, ensuring proper conformation and activity [50]. There are no published data to date of the impact of rutin on the level of HSP70; however, this polyphenol has been found as the one that increases expression of another chaperone, HSP90 [36]. Additionally, as was shown in this study, only rutin significantly enhanced active HPS90 complex formation. On the other hand, ascorbic acid when used alone has no significant effect on the HSP70 and HSP90 levels both in this study and in previous publications concerning other cell types (heart, liver) [51,52]. Only the combination of ascorbic acid and rutin treatment causes a significant increase in HSP70 expression, ensuring functional conformation of proteins, even under UV-induced oxidative stress conditions.

Ascorbic acid and rutin used separately, and in combination, have a strong antioxidant potential and significantly decrease the expression of antioxidant proteins such as thioredoxin and glutaredoxin in both UV-irradiated and not-irradiated cells. In the case of thioredoxin, similar results are observed for skin fibroblasts cultured in a monolayer [32]. Polyphenols, including rutin, are known for their inhibitory effect on thioredoxin reductase, an enzyme that restores the level of reduced thioredoxin in cells under oxidative stress [53]. Therefore, rutin in cooperation with ascorbic acid may support the cellular antioxidant system in a manner where the cells expend no energy to maintain a high level of endogenous antioxidants in the cytoplasm. Moreover, similar changes are observed in the case of glutaredoxin, which reduces oxidized proteins [54]. The oxidized form of glutaredoxin is non-enzymatically reduced by glutathione, which drives the action of the glutathione system, including glutathione reductase [55]. On the other hand, glutaredoxin enzymatically reduces ascorbic acid under oxidative stress conditions [56], which in rutin treated cells may further promote the cytoprotection seen in cells treated with ascorbic acid-rutin combination.

The antioxidant capacity of ascorbic acid and rutin, in parallel with their induced stimulation of antioxidant proteins, significantly reduces UV-induced oxidative stress, thereby reducing the oxidative metabolism of lipids and lowering the level of reactive lipid peroxidation products [32]. This may be the reason for the prevention of protein adduct formation with lipid peroxidation products observed in this study. Cells treated with ascorbic acid and rutin together strongly prevent the formation of 4-ONE-protein adducts [6]. Moreover, adduct formation in ascorbic acid and rutin treated, UV-irradiated fibroblasts involve molecules with different functions. As a result, UVA- and UVB-irradiated cells are characterized by modified proteins with catalytic and molecular transducer activity and transporters, which disrupt cellular metabolism and thereby reduce skin cell function [57]. However, the protective action of ascorbic acid and rutin in combination causes protein modifications by production lipid peroxidation products that include impacts on structural proteins, which protect proteins with enzymatic activity and ensure that cells function under oxidative stress conditions.

Cells treated with rutin alone show changes in their protein profile by the rutin-protein adducts formation [36]. As shown in this study, the main proteins modified by rutin in 3D cultured fibroblasts, regardless of cell treatment, are FERM and PDZ domain-containing protein 1 (Q5SYB0). This protein set is primarily responsible for the establishment of the localization of other proteins to membranes, and also receptors involved in the regulation of the G protein-coupled signaling pathway connected with cell-cell interactions [58]. Moreover, in UV-irradiated cells, rutin through adducts creation can affect the activity of regulators of G-protein signaling (RGS12/22) (Q56A82, Q8NE09). Most of the proteins from the RGS family in their structure, similar to FERM and PDZ domain-containing protein 1, contain PDZ domains [59]. Independent of that, the presence of the RGS box in their molecules provides a specific interaction with the α subunits of tye G proteins and enhances the GTPase activities [60]. Therefore, regulators of G-protein signaling are involved in the various biological processes stimulation; however, dysfunctions in the functioning of RGS12 or RGS22 are conducive to the development of tumors [61,62].

On the other hand, UV radiation stimulates rutin antioxidant action by creating adducts with Keap1 (Q14145) at cysteine-273. This amino acid residue is one of the three cysteines responsible for proper Keap1 conformation and biological activity associated with Nrf2 binding and degradation [63]. The oxidation of one or all of these cysteine residues causes Keap1 to be unable to bind and ubiquitinate Nrf2 for degradation; instead, Nrf2 can be activated by phosphorylation and translocated to the nucleus where it can bind to the DNA at ARE elements to initiate transcription [36,64,65]. The same modification of Keap1 has been previously observed in the case of this protein incubated with rutin following UVB irradiation [36]. Moreover, it was previously shown that rutin, because of its polyphenolic component quercetin, may be able to interact with Keap1 in another part of this molecule—with cysteine residue at Nrf2-binding sites, and because of that, it leads to Keap1 ubiquitination [66] On the other hand, using the same oxidative conditions, ascorbic acid alone leads to decreased Nrf2 levels [67]; however, by stimulating the penetration of rutin through cell membranes, it additionally promotes the formation of rutin-Keap1 adducts.

Following UVB irradiation, rutin is also bonded to lysine-specific histone demethylase, Q8NB78, and, by disrupting the structure of this protein, may ensure high levels of histone methylation and, hence, transcriptional activity, which was previously observed in the case of other natural polyphenols, including quercetin [68]. As a result, cytoprotective effects are observed on UV-irradiated cells. On the other hand, rutin modification of telomeres by protein 1 (POT1, H7C4C7) may influence the process of telomere elongation. However, the mechanism of rutin action on POT1 as a negative regulator of telomerase activity [69] is not known. Some recent publications for other polyphenols, such as epigallocatechin gallate, show that compounds of this nature may inhibit telomere shortening or cause fragmentation depending on the cell type [70,71]. Despite this, increased rutin-protein adduct levels observed in cells treated with a combination of rutin and ascorbic acid might be connected with ascorbic acid assisted rutin penetration into the cell cytoplasm, as well as rutin protection against oxidation in physically stressed fibroblasts [32,72].

The data presented in this study show that rutin and ascorbic acid, used in combination, have the potential to prevent UV-induced overexpression of the proteins involved in DNA organization and expression and protein biosynthesis to a higher degree than is observed when using either compound alone. Moreover, the antioxidant properties of these compounds protect skin fibroblasts against oxidative stress and significantly prevent protein modification by lipid peroxidation products, suggesting that ascorbic acid and rutin in combination are an effective team in skin protection against damage caused by UV radiation.

## Supplementary Materials

The following are available online at https://www.mdpi.com/2072-6643/12/4/1074/s1, Table S1: List of proteins indicated in the 3D cultured fibroblasts exposed to UVB irradiation (200 mJ/cm2) and treated with ascorbic acid [100 µM] or/and rutin [25 µM].
