# Supplementary material for: Cytoprotective Effect of Ascorbic Acid and Rutin against Oxidative Changes in the Proteome of Skin Fibroblasts Cultured in a Three-Dimensional System
Source: Nutrients. 2020 Apr 13;12(4):1074. doi: 10.3390/nu12041074 (PMC7230807; doi:10.3390/nu12041074)
Supplement: Supplementary file 1 [file nutrients-12-01074-s001.pdf]

Supplementary table S1.
List of proteins indicated in the 3D cultured fibroblasts exposed to UVB irradiation (200 mJ/cm2) and treated with ascorbic acid [100 µM] or/and rutin [25 µM]. Mean values of three independent label free experiments are presented. Abbreviations: Asc, ascorbic acid; Ctr, control; Rut, rutin; N/D, not detected;

ID	Ctr	Asc	Rut	Asc + Rut	UVB	UVB + Asc	UVB + Rut	UVB + Asc + Rut
A0A024QZ64	7266666.67	5633333.33	366.67	1666666.67	8433333.33	12333333.33	10166666.67	24000000.00
A0A024QZK7	N/D	N/D	N/D	N/D	1900000.00	100.00	2233333.33	N/D
A0A024QZK8	N/D	N/D	N/D	N/D	600000.00	N/D	N/D	1300000.00
A0A024QZN9	N/D	N/D	N/D	850000.00	3266666.67	3400000.00	1156666.67	1000.00
A0A024QZT0	N/D	N/D	N/D	850000.00	3266666.67	3400000.00	1156666.67	1000.00
A0A024QZT9	N/D	N/D	N/D	580000.00	1900000.00	990000.00	N/D	2400000.00
A0A024QZX5	2000000.00	1006666.67	N/D	706666.67	253366.67	3800000.00	580000.00	3500000.00
A0A024QZZ7	N/D	4400000.00	N/D	5600000.00	3366666.67	2466666.67	4400000.00	16000000.00
A0A024R0E5	N/D	N/D	N/D	N/D	N/D	2600000.00	4400000.00	6666666.67
A0A024R0J9	N/D	N/D	N/D	N/D	4566666.67	N/D	N/D	N/D
A0A024R1N4	N/D	1200000.00	N/D	490000.00	8300000.00	3900000.00	4900000.00	6200000.00
A0A024R1Y2	N/D	N/D	N/D	1200000.00	1500000.00	2800000.00	4033333.33	N/D
A0A024R228	N/D	880000.00	2300000.00	2000000.00	N/D	4666666.67	N/D	300000.00
A0A024R231	3133333.33	4700000.00	N/D	5800000.00	8400000.00	7800000.00	400.00	800.00
A0A024R280	N/D	1466666.67	N/D	N/D	460100.00	2300000.00	1200066.67	1600000.00
A0A024R2P0	N/D	1140000.00	400533.33	1900000.00	15000000.00	6800000.00	N/D	800.00
A0A024R2Q4	N/D	420000.00	N/D	N/D	N/D	N/D	N/D	90.00
A0A024R382	N/D	N/D	N/D	N/D	4266666.67	1700000.00	3033333.33	N/D
A0A024R3W7	N/D	N/D	N/D	N/D	550000.00	N/D	N/D	N/D
A0A024R3X7	N/D	7600000.00	6400000.00	9400000.00	9666666.67	800.00	1666666.67	54000000.00
A0A024R3Z6	N/D	N/D	N/D	N/D	300.00	3400000.00	1833333.33	N/D
A0A024R451	N/D	N/D	N/D	N/D	1500000.00	200.00	1566666.67	N/D
A0A024R498	N/D	N/D	N/D	N/D	1500000.00	200.00	1566666.67	N/D
A0A024R592	N/D	N/D	N/D	N/D	12666666.67	4966666.67	1333.33	4900000.00
A0A024R5W6	N/D	N/D	N/D	N/D	3266666.67	N/D	N/D	N/D
A0A024R5Z7	3333333.33	3233333.33	3600000.00	4266666.67	2000000.00	14666666.67	5100000.00	3000.00
A0A024R5Z8	N/D	N/D	N/D	N/D	100.00	N/D	N/D	3500000.00
A0A024R609	12866666.67	6233333.33	3900000.00	8966666.67	52766666.67	35000000.00	43166666.67	3000.00
A0A024R6I7	3166666.67	2400000.00	N/D	2660000.00	N/D	3200000.00	3833333.33	3666666.67
A0A024R6W2	N/D	N/D	N/D	N/D	640000.00	3700000.00	N/D	400.00
A0A024R718	8933333.33	5233333.33	7400000.00	5600000.00	24576666.67	16666666.67	24066666.67	13000000.00
A0A024R7F7	N/D	N/D	N/D	N/D	3000000.00	N/D	N/D	60.00
A0A024R7M0	N/D	N/D	N/D	N/D	1296666.67	N/D	N/D	1666666.67
A0A024R7T3	N/D	N/D	200.00	N/D	2966666.67	2200000.00	N/D	N/D
A0A024R814	N/D	480000.00	N/D	N/D	2660000.00	1800000.00	840000.00	3200000.00
A0A024R944	N/D	N/D	1600000.00	N/D	N/D	2600000.00	1266666.67	N/D
A0A024R9E2	N/D	N/D	N/D	N/D	6366666.67	N/D	400.00	N/D
A0A024RA28	N/D	5200000.00	1800000.00	11266666.67	1666666.67	2700000.00	1566666.67	9200000.00
A0A024RA52	N/D	1426666.67	N/D	3800000.00	2420000.00	6800000.00	2566666.67	9300000.00
A0A024RA55	N/D	1466666.67	1533333.33	N/D	N/D	N/D	2833333.33	N/D
A0A024RAD5	N/D	N/D	N/D	N/D	2966666.67	2000000.00	3333333.33	N/D
A0A024RAI1	N/D	N/D	N/D	N/D	6266666.67	2400000.00	3833333.33	N/D
A0A024RAM0	N/D	N/D	N/D	N/D	3000000.00	1900000.00	N/D	60.00
A0A024RAM2	N/D	880000.00	4200000.00	1200000.00	1416666.67	3900000.00	880000.00	6666666.67
A0A024RAZ7	N/D	2300100.00	1800000.00	2033400.00	3266666.67	6466666.67	966733.33	24000000.00
A0A024RB53	N/D	2300100.00	1800000.00	2033400.00	3266666.67	6466666.67	966733.33	24000000.00
A0A024RB85	N/D	N/D	N/D	N/D	2200000.00	N/D	2133333.33	2500000.00
A0A024RB99	N/D	N/D	N/D	N/D	3566666.67	N/D	4000000.00	N/D
A0A024RBK9	4400000.00	5133333.33	6800000.00	6500000.00	14766666.67	1266666.67	21566666.67	3300000.00
A0A024RBS2	N/D	800000.00	N/D	866666.67	N/D	3200000.00	800000.00	7066666.67
A0A024RC87	N/D	N/D	N/D	N/D	3666666.67	1400000.00	3933333.33	N/D
A0A024RD93	N/D	N/D	N/D	N/D	3333333.33	1600000.00	N/D	N/D
A0A024RDB4	N/D	3600000.00	N/D	600200.00	900.00	3000000.00	3933333.33	840000.00
A0A024RDF4	N/D	3600000.00	N/D	600200.00	900.00	3000000.00	4266666.67	840000.00
A0A024RDQ0	N/D	N/D	N/D	N/D	3566666.67	2000000.00	133.33	N/D
A0A024RDR0	N/D	1700000.00	N/D	2800000.00	1620000.00	2700000.00	2033333.33	3600000.00
A0A075B7A0	N/D	N/D	N/D	N/D	266.67	1900000.00	600000.00	N/D
A0A087WUI2	N/D	4100000.00	1933333.33	600466.67	1766666.67	5066666.67	1566666.67	9200000.00
A0A087WUS4	N/D	N/D	N/D	N/D	3966666.67	N/D	4466666.67	N/D
A0A087WUV8	2466666.67	N/D	N/D	2666666.67	6666666.67	2933333.33	3220000.00	490000.00
A0A087WVQ9	N/D	N/D	3700000.00	N/D	13666666.67	4700000.00	3400000.00	1000.00
A0A087WWU8	N/D	N/D	N/D	N/D	3033333.33	N/D	N/D	N/D
A0A087WWY3	166.67	N/D	N/D	N/D	100.00	166.67	4566666.67	N/D
A0A087WXQ8	870000.00	1700000.00	700.00	2933333.33	1666666.67	6556666.67	4233333.33	N/D
A0A087WYC5	2600000.00	2300000.00	N/D	4400000.00	12666666.67	4200000.00	5066666.67	N/D
A0A087WYJ9	N/D	N/D	N/D	N/D	4333333.33	N/D	3666666.67	N/D
A0A087WYS6	N/D	3300000.00	N/D	2600000.00	3033333.33	8800000.00	4033333.33	24000000.00
A0A087WYY5	N/D	1666666.67	1800000.00	833533.33	N/D	3300000.00	2133333.33	N/D
A0A087WYZ1	N/D	N/D	N/D	N/D	N/D	1700000.00	4266666.67	3966666.67
A0A087X027	N/D	N/D	N/D	100.00	3933333.33	2900000.00	N/D	N/D
A0A087X079	2600000.00	2300000.00	N/D	4400000.00	12666666.67	4200000.00	5066666.67	N/D
A0A087X1B9	2400000.00	1400000.00	N/D	2300000.00	1600000.00	3300000.00	3766666.67	N/D
A0A087X1N8	2000000.00	1006666.67	N/D	706666.67	253366.67	3800000.00	580000.00	3500000.00
A0A087X1V9	N/D	N/D	9800000.00	5200000.00	3300000.00	466.67	N/D	30000000.00
A0A087X297	N/D	N/D	N/D	2666666.67	533.33	400.00	5900000.00	2200000.00
A0A087X2B5	2533333.33	1300000.00	N/D	2666666.67	6666666.67	3600000.00	3720000.00	490000.00
A0A087X2C0	N/D	N/D	N/D	N/D	4333333.33	N/D	3666666.67	N/D
A0A087X2I4	N/D	40.00	N/D	N/D	N/D	5566666.67	40.00	N/D
A0A090N8G0	N/D	1700000.00	N/D	N/D	867000.00	400.00	5500000.00	N/D
A0A0A0MS07	2600000.00	2300000.00	N/D	4400000.00	1000.00	5066666.67	5066666.67	N/D

A0A0A0MS08	2600000.00	2300000.00	N/D	4400000.00	12666666.67	4200000.00	5066666.67	N/D
A0A0A0MS42	N/D	N/D	N/D	N/D	N/D	1466666.67	N/D	N/D
A0A0A0MSB2	933333.33	1800000.00	8200000.00	3000000.00	2666666.67	5176666.67	5333400.00	4000000.00
A0A0A0MSI0	2266666.67	3353333.33	9600000.00	800000.00	8200000.00	6533333.33	5700000.00	42000000.00
A0A0A0MSK8	N/D	N/D	9700000.00	N/D	1800000.00	3600000.00	1533333.33	N/D
A0A0A0MSX9	N/D	N/D	N/D	N/D	3366666.67	2900000.00	2733333.33	N/D
A0A0A0U6N4	3766666.67	1700000.00	14000000.00	3500000.00	3000.00	9166666.67	21333333.33	6966666.67
A0A0A6YYG9	N/D	1700000.00	930000.00	2000000.00	2933333.33	2700000.00	936666.67	5300000.00
A0A0C4DGC5	N/D	2433333.33	N/D	3566666.67	3000000.00	12000000.00	2700000.00	24000000.00
A0A0C4DGH5	N/D	N/D	N/D	N/D	1300000.00	N/D	2033333.33	N/D
A0A0C4DGS1	N/D	N/D	N/D	N/D	2966666.67	2000000.00	3666666.67	N/D
A0A0G2JNK4	2600000.00	2300000.00	N/D	4400000.00	12666666.67	4200000.00	5066666.67	N/D
A0A0G2JPD4	N/D	N/D	N/D	N/D	3933333.33	N/D	N/D	N/D
A0A0G2JRN3	3166666.67	2400000.00	N/D	2600000.00	N/D	3200000.00	3833333.33	3666666.67
A0A0K0K1I0	N/D	N/D	N/D	N/D	3266666.67	N/D	N/D	N/D
A0A0S2Z3M4	N/D	N/D	N/D	N/D	590000.00	1800000.00	N/D	1200000.00
A0A0S2Z3S2	N/D	406700.00	N/D	3200000.00	4333333.33	2300000.00	N/D	N/D
A0A0S2Z3X3	N/D	N/D	N/D	N/D	N/D	N/D	4366666.67	N/D
A0A0S2Z4G7	N/D	1466666.67	8900000.00	900.00	2400000.00	7700000.00	2766666.67	8500000.00
A0A0S2Z4G8	N/D	N/D	N/D	N/D	3033333.33	N/D	N/D	N/D
A0A0S2Z4J1	N/D	100.00	100.00	2400000.00	800000.00	4400000.00	133.33	N/D
A0A0S2Z4W7	N/D	N/D	N/D	N/D	3566666.67	N/D	N/D	N/D
A0A0S2Z5C0	4233333.33	466.67	6200000.00	4000000.00	6966666.67	5833333.33	4800000.00	6966666.67
A1L185	N/D	N/D	N/D	N/D	N/D	930000.00	4266666.67	4566666.67
A4D2J6	N/D	466.67	2800000.00	3673333.33	1253333.33	7400000.00	1566666.67	15000000.00
A4QPB0	2900000.00	1366666.67	N/D	6400000.00	13666666.67	3766666.67	6233333.33	N/D
A4UCS5	N/D	N/D	N/D	N/D	1266666.67	N/D	N/D	N/D
A4UCS6	N/D	2033333.33	N/D	2733333.33	273600.00	1000.00	366.67	15000000.00
A4UCT1	28000000.00	28666666.67	21433333.33	53500000.00	89633333.33	190000000.00	42666666.67	100766666.67
A6NFX8	N/D	N/D	N/D	N/D	1400000.00	3000000.00	N/D	3666666.67
A6NIW5	N/D	2016666.67	893333.33	4400000.00	1143333.33	8000000.00	1566666.67	7466666.67
A6NJU6	N/D	N/D	N/D	N/D	1366666.67	N/D	N/D	3666666.67
A6NLN1	N/D	1800000.00	2600000.00	N/D	3300000.00	6200000.00	2133333.33	3200000.00
A6PVX1	N/D	N/D	N/D	N/D	N/D	1600000.00	1333.33	N/D
A6YP93	N/D	N/D	N/D	N/D	1366666.67	N/D	N/D	N/D
A8K3C3	N/D	N/D	1900000.00	2000000.00	3033333.33	300.00	1120000.00	N/D
A8K3Q7	N/D	N/D	N/D	N/D	3266666.67	N/D	2733333.33	N/D
A8K401	N/D	N/D	N/D	N/D	1266666.67	100.00	N/D	6200000.00
A8K482	2366666.67	7900000.00	4400000.00	7166666.67	4786666.67	500000.00	6190000.00	2600000.00
A8K4W0	N/D	N/D	N/D	N/D	830000.00	100.00	N/D	4800000.00
A8K4Y7	166.67	10166666.67	10600000.00	6333333.33	600.00	800.00	1666766.67	94000000.00
A8K4Z4	N/D	800000.00	N/D	866666.67	N/D	3200000.00	800000.00	7066666.67
A8K6V6	2366666.67	1010000.00	N/D	3200000.00	5000000.00	2766666.67	1053466.67	N/D
A8K7J7	N/D	N/D	N/D	N/D	1900000.00	100.00	2233333.33	N/D
A8K8D9	1966933.33	6000000.00	3400000.00	800266.67	23400000.00	19966666.67	37000000.00	1000.00
A8K9C4	N/D	N/D	3700000.00	N/D	13666666.67	4700000.00	3400000.00	1000.00
A8K9J7	N/D	4400000.00	N/D	5600000.00	3366666.67	2466666.67	4400000.00	16000000.00
A8MT02	N/D	2300000.00	7200000.00	1900000.00	2933333.33	3000000.00	233.33	6866666.67
A8MTM1	N/D	N/D	N/D	N/D	2933333.33	200.00	N/D	14000000.00
A8MVZ9	7266666.67	5633333.33	366.67	1666666.67	8433333.33	12333333.33	10166666.67	24000000.00
B0YIW6	N/D	N/D	N/D	100.00	N/D	1300000.00	N/D	N/D
B0YJ74	N/D	3300000.00	N/D	2500000.00	3033333.33	6900000.00	4033333.33	16000000.00
B0YJC4	3933333.33	2400000.00	3000000.00	706666.67	7466666.67	N/D	2066833.33	N/D
B0YJC5	1866666.67	1700000.00	2700000.00	N/D	3800000.00	N/D	2766666.67	N/D
B1ANR0	N/D	N/D	N/D	N/D	3366666.67	N/D	N/D	N/D
B1AP13	2933333.33	1133400.00	N/D	N/D	12666666.67	2700000.00	2233333.33	N/D
B1AP15	3333333.33	3400000.00	N/D	N/D	7266666.67	4300000.00	1833333.33	N/D
B2R4R0	4666666.67	24200000.00	54433333.33	28600000.00	20966666.67	1772000.00	21333333.33	280866666.67
B2R5B3	N/D	1400000.00	N/D	5800000.00	8900000.00	N/D	1733333.33	56666666.67
B2R6A7	N/D	N/D	N/D	N/D	3300000.00	200.00	N/D	N/D
B2R6Y1	166.67	10166666.67	10600000.00	6333333.33	600.00	800.00	1666766.67	94000000.00
B2RAQ9	N/D	N/D	N/D	N/D	N/D	4966666.67	N/D	N/D
B2RD14	N/D	2000000.00	1533333.33	2133333.33	8666666.67	7400000.00	N/D	8200000.00
B2RDG0	N/D	1466666.67	3533333.33	2600000.00	8900000.00	4866666.67	2133333.33	1000.00
B2RE56	200.00	1167333.33	2000.00	6800000.00	10066666.67	14666733.33	4100000.00	94000000.00
B3GQE5	N/D	N/D	9700000.00	N/D	1800000.00	3600000.00	1533333.33	N/D
B3KPA6	N/D	N/D	N/D	N/D	3300000.00	3200000.00	N/D	N/D
B3KQT2	2100000.00	200.00	1200000.00	1200000.00	15000000.00	5166666.67	32433333.33	6600000.00
B3KQV6	N/D	N/D	N/D	N/D	266.67	1700000.00	4400000.00	N/D
B3KRM8	N/D	N/D	N/D	N/D	980000.00	N/D	N/D	4300000.00
B3KSI4	5800000.00	18000000.00	8300000.00	11500066.67	2934333.33	9600000.00	13500000.00	12240000.00
B3KSJ6	3766666.67	N/D	N/D	2000000.00	16666666.67	600.00	17000000.00	N/D
B3KT93	N/D	N/D	N/D	N/D	6366666.67	N/D	400.00	N/D
B3KTA3	313380.00	2200000.00	4900000.00	2666666.67	4986666.67	3443333.33	5066666.67	4300000.00
B3KTM1	1966666.67	6466666.67	17000000.00	4000333.33	2220000.00	16500000.00	2400000.00	29000000.00
B3KTM6	N/D	N/D	N/D	N/D	9833333.33	2600000.00	133.33	4000000.00
B3KWE1	N/D	N/D	N/D	N/D	3900000.00	N/D	4800000.00	N/D
B3KXY9	N/D	N/D	N/D	N/D	1900000.00	100.00	2233333.33	N/D
B3VMW0	4600000.00	4433333.33	6800000.00	277333.33	600.00	7233333.33	4766666.67	N/D
B4DEB1	166.67	10166666.67	10600000.00	6333333.33	600.00	800.00	1666766.67	94000000.00
B4DEV8	N/D	470000.00	6200000.00	1666666.67	3266666.67	3466666.67	470000.00	9300000.00
B4DHB3	4133333.33	9500000.00	10666800.00	5066666.67	50000200.00	33533333.33	2870000.00	39000000.00
B4DHQ3	N/D	1466666.67	N/D	N/D	2200100.00	2300000.00	1200066.67	1600000.00
B4DHZ6	400.00	N/D	300.00	4666666.67	3566666.67	400.00	N/D	N/D
B4DID5	N/D	N/D	3300000.00	1666666.67	3166666.67	5066666.67	N/D	7466666.67
B4DJ63	N/D	N/D	N/D	N/D	3566666.67	N/D	4000000.00	N/D

B4DJ75	N/D	866733.33	1800000.00	833533.33	1086666.67	3600000.00	2133333.33	N/D
B4DJC3	N/D	N/D	N/D	N/D	N/D	1300000.00	N/D	6666666.67
B4DJQ3	N/D	N/D	N/D	N/D	3566666.67	N/D	3033333.33	N/D
B4DK69	N/D	N/D	2700000.00	2500000.00	696666.67	5366666.67	N/D	13000000.00
B4DM74	N/D	3000000.00	6300000.00	3500000.00	1366666.67	2466666.67	600.00	3800000.00
B4DM82	200.00	4366666.67	6133333.33	5433333.33	1068666.67	733.33	2666733.33	43000000.00
B4DMK9	2366666.67	2000000.00	N/D	2500000.00	393433.33	3366666.67	3866666.67	1200000.00
B4DR52	N/D	4400000.00	N/D	5600000.00	3366666.67	2466666.67	4400000.00	16000000.00
B4DRT3	9866666.67	1300333.33	3800000.00	4200000.00	32866766.67	23333333.33	34233333.33	3000.00
B4DSU6	N/D	5700000.00	6400066.67	3500000.00	1500066.67	14666666.67	2266733.33	35000000.00
B4DV51	N/D	2500000.00	1866666.67	1566666.67	500.00	6000000.00	1853333.33	7333333.33
B4DVJ0	4400000.00	1666690.00	5066666.67	1487000.00	15460000.00	13866666.67	1334666.67	4133333.33
B4DVS0	N/D	2300000.00	7200000.00	1900000.00	2933333.33	3000000.00	233.33	6866666.67
B4DWA7	N/D	N/D	N/D	N/D	3566666.67	N/D	4000000.00	N/D
B4DWQ3	6766666.67	12566666.67	1566666.67	4000466.67	136666666.67	32500000.00	1633466.67	35000000.00
B4DX99	N/D	N/D	N/D	N/D	4566666.67	930000.00	880000.00	N/D
B4DXW1	N/D	N/D	N/D	N/D	N/D	2400000.00	4166666.67	6266666.67
B4DYA7	1966933.33	6000000.00	3400000.00	800266.67	22400000.00	19966666.67	31000000.00	N/D
B4E0X6	N/D	470000.00	6200000.00	1666666.67	3266666.67	3466666.67	470000.00	9300000.00
B4E1G2	N/D	N/D	N/D	N/D	3566666.67	N/D	4000000.00	N/D
B4E1H9	9100000.00	14533333.33	12300000.00	9333333.33	1806766.67	54633333.33	53433333.33	50000000.00
B4E380	166.67	10166666.67	10600000.00	6333333.33	600.00	800.00	1666766.67	94000000.00
B5BUB5	N/D	N/D	800000.00	N/D	3566666.67	2200000.00	133.33	N/D
B5MCX3	N/D	N/D	N/D	N/D	2666666.67	N/D	N/D	1666666.67
B5MDF5	N/D	2900000.00	5133333.33	2300000.00	5500000.00	1000.00	1933333.33	22000000.00
B7Z2F4	N/D	N/D	1900000.00	2000000.00	3033333.33	300.00	1120000.00	N/D
B7Z478	N/D	2800033.33	4333333.33	1223600.00	866766.67	9000000.00	4400000.00	N/D
B7Z4B8	N/D	N/D	N/D	N/D	4566666.67	N/D	N/D	N/D
B7Z514	2266666.67	N/D	N/D	N/D	3366666.67	1200000.00	2833333.33	N/D
B7Z596	N/D	N/D	N/D	N/D	3266666.67	N/D	N/D	N/D
B7Z645	N/D	N/D	1533333.33	N/D	N/D	4966666.67	7900000.00	9666666.67
B7Z6S9	N/D	N/D	N/D	N/D	3300000.00	200.00	N/D	N/D
B7Z722	N/D	N/D	N/D	N/D	3266666.67	N/D	N/D	N/D
B7Z9L0	N/D	N/D	1900000.00	2000000.00	3033333.33	300.00	1120000.00	N/D
B7ZB67	N/D	866733.33	1800000.00	833533.33	1086666.67	3600000.00	2133333.33	N/D
B8Y0L3	N/D	N/D	9700000.00	N/D	2666666.67	3600000.00	1533333.33	N/D
B8ZZ54	N/D	5366666.67	6400000.00	1400600.00	9666666.67	5466666.67	1700000.00	26000000.00
B8ZZA3	N/D	N/D	N/D	2666666.67	533.33	400.00	5900000.00	2200000.00
B9VP24	N/D	N/D	N/D	N/D	N/D	N/D	3133333.33	1000.00
C9IZA5	N/D	N/D	200.00	N/D	600000.00	N/D	2733333.33	N/D
C9J4V0	N/D	100.00	2300000.00	1400000.00	1500000.00	2466666.67	133.33	4800000.00
C9J7D1	N/D	100.00	2300000.00	1400000.00	1500000.00	2466666.67	133.33	4800000.00
C9J7H8	2433333.33	8600000.00	9800000.00	9000000.00	4766666.67	14166666.67	4266666.67	46000000.00
C9JCK5	N/D	2000000.00	N/D	2000000.00	N/D	6800000.00	3033333.33	9300000.00
C9JF79	N/D	N/D	N/D	N/D	1366666.67	2900000.00	N/D	2000000.00
C9JFC0	933333.33	1800000.00	8200000.00	3000000.00	2666666.67	5176666.67	5333400.00	4000000.00
C9JH19	N/D	N/D	N/D	1600000.00	6000000.00	6300000.00	N/D	1156666.67
C9JI87	N/D	N/D	N/D	N/D	1366666.67	N/D	1700000.00	4800000.00
C9JIJ5	N/D	N/D	N/D	N/D	830000.00	1800000.00	840000.00	5300000.00
C9JL25	N/D	N/D	8500000.00	N/D	4466666.67	N/D	3666666.67	N/D
C9JRZ8	1046666.67	9600000.00	12000000.00	10666666.67	8366666.67	24433333.33	4900000.00	63000000.00
C9JV77	6433333.33	2400000.00	3580000.00	243766.67	3966666.67	3633333.33	2833333.33	N/D
C9JW96	N/D	N/D	N/D	N/D	1266666.67	100.00	N/D	6200000.00
C9JZ20	N/D	N/D	N/D	N/D	1266666.67	100.00	N/D	6200000.00
C9JZ88	N/D	N/D	N/D	N/D	830000.00	2200000.00	840000.00	5300000.00
C9K0U8	N/D	1400000.00	6033333.33	2333333.33	656666.67	3200000.00	1733333.33	500.00
D3DPU2	N/D	N/D	N/D	N/D	N/D	1400000.00	N/D	N/D
D3DV26	N/D	4300000.00	N/D	3000000.00	13000000.00	9300000.00	4366666.67	N/D
D3DV87	N/D	N/D	N/D	N/D	3300000.00	200.00	N/D	N/D
D6R9P3	N/D	1800000.00	N/D	N/D	9200000.00	2300000.00	2133333.33	3066666.67
D6RAU2	N/D	N/D	N/D	516666.67	1500000.00	3000000.00	N/D	N/D
D6RBL5	466800.00	3133333.33	3533333.33	2700000.00	3933333.33	8433333.33	934000.00	26000000.00
D6RBZ0	N/D	1800000.00	N/D	N/D	9200000.00	2300000.00	2133333.33	3066666.67
D6RCN3	N/D	N/D	N/D	N/D	3033333.33	N/D	N/D	8300000.00
D6RD18	N/D	646666.67	N/D	N/D	1686666.67	1400000.00	3666666.67	990000.00
D6RF23	2000000.00	2400000.00	N/D	N/D	653400.00	4966666.67	2733333.33	4400000.00
D6RF53	N/D	1400000.00	1533333.33	1600000.00	3166666.67	2600000.00	1733333.33	8000000.00
D6RG13	N/D	N/D	N/D	N/D	830000.00	100.00	N/D	4800000.00
D9YZV5	N/D	N/D	N/D	N/D	3266666.67	N/D	N/D	N/D
E5RG29	N/D	N/D	9700000.00	N/D	1800000.00	3600000.00	1533333.33	N/D
E5RHP0	400466.67	5866666.67	11800000.00	7333366.67	13666666.67	16400000.00	3833433.33	40000000.00
E5RI98	N/D	1466666.67	8900000.00	9200000.00	3100000.00	7300000.00	3066666.67	16000000.00
E5RJB9	N/D	N/D	N/D	N/D	4466666.67	N/D	366.67	N/D
E7EN95	N/D	N/D	N/D	N/D	2666666.67	N/D	6500000.00	5333333.33
E7EPJ9	3666666.67	2033333.33	9900000.00	N/D	13666666.67	12000000.00	3733333.33	13000000.00
E7EQB2	4600000.00	4433333.33	6800000.00	277333.33	600.00	7233333.33	4766666.67	N/D
E7EQG2	2100000.00	980000.00	1600000.00	N/D	453533.33	3700000.00	5133333.33	5300000.00
E7EQR4	N/D	N/D	N/D	N/D	6000000.00	5133333.33	600.00	N/D
E7EQV9	N/D	420000.00	N/D	N/D	N/D	N/D	N/D	90.00
E7ER27	N/D	100.00	100.00	2400000.00	800000.00	4400000.00	133.33	N/D
E7ER44	4600000.00	4433333.33	6800000.00	277333.33	600.00	7233333.33	4766666.67	N/D
E7ER95	N/D	N/D	N/D	N/D	2800000.00	7800000.00	7900000.00	7900000.00
E7ESE2	N/D	N/D	N/D	N/D	1266666.67	100.00	N/D	6200000.00
E7ESH4	N/D	N/D	4533333.33	N/D	13000000.00	N/D	3766666.67	N/D
E7ESK7	N/D	2566666.67	1200000.00	2053333.33	5600000.00	600.00	966733.33	1000.00
E7ET17	N/D	100.00	100.00	2400000.00	N/D	4400000.00	133.33	N/D

E7ETD8	N/D	866733.33	1800000.00	833533.33	N/D	3600000.00	2133333.33	N/D
E7ETE2	N/D	N/D	N/D	N/D	3900000.00	N/D	4800000.00	N/D
E7ETZ0	N/D	N/D	N/D	N/D	N/D	N/D	1933333.33	N/D
E7EUI8	1966933.33	5266666.67	3333333.33	800266.67	22400000.00	19966666.67	31000000.00	N/D
E7EUY5	N/D	1400000.00	6033333.33	2333333.33	656666.67	3200000.00	1733333.33	500.00
E7EX29	N/D	2566666.67	1200000.00	2053333.33	5600000.00	600.00	966733.33	1000.00
E7EXB4	N/D	N/D	4533333.33	N/D	9900000.00	N/D	3500000.00	N/D
E9PCW0	N/D	N/D	N/D	N/D	1266666.67	100.00	N/D	6200000.00
E9PCX2	N/D	4466666.67	1866933.33	2666666.67	N/D	7300000.00	4033333.33	6666666.67
E9PD14	N/D	N/D	N/D	516666.67	1500000.00	3000000.00	N/D	N/D
E9PD92	1933600.00	5033333.33	2933333.33	800266.67	22400000.00	19933333.33	31000000.00	N/D
E9PEX6	2366666.67	2000000.00	N/D	2500000.00	253433.33	3366666.67	3866666.67	1200000.00
E9PGT1	N/D	N/D	N/D	N/D	980000.00	N/D	N/D	4300000.00
E9PJH4	N/D	N/D	N/D	N/D	1300000.00	N/D	450000.00	7333333.33
E9PK54	20666666.67	6133333.33	19233333.33	800166.67	3666.67	9800000.00	19300000.00	14000000.00
E9PK82	N/D	N/D	N/D	N/D	1300000.00	N/D	450000.00	7333333.33
E9PKD3	2633333.33	2800000.00	8800000.00	3500000.00	26666666.67	833866.67	9000000.00	N/D
E9PLF4	20666666.67	6133333.33	19233333.33	800166.67	3666.67	9800000.00	19300000.00	14000000.00
E9PLK3	1333333.33	1600000.00	N/D	1400000.00	590000.00	2266666.67	6400000.00	N/D
E9PMD7	N/D	1666666.67	1800000.00	833533.33	1086666.67	3300000.00	2133333.33	N/D
E9PN89	4866900.00	1667200.00	17966666.67	5100000.00	41666666.67	8666900.00	14666966.67	6866666.67
E9PNH1	N/D	N/D	N/D	N/D	3300000.00	N/D	2233333.33	N/D
E9PPH5	N/D	N/D	N/D	N/D	2200033.33	N/D	N/D	3200000.00
E9PPY6	N/D	100.00	N/D	N/D	26666666.67	400.00	8233333.33	N/D
E9PQ96	N/D	N/D	N/D	N/D	1300000.00	N/D	450000.00	7333333.33
E9PQQ4	20666666.67	6133333.33	19233333.33	800166.67	3666.67	9800000.00	19300000.00	14000000.00
E9PRK8	2226666.67	1433333.33	N/D	3000100.00	2933333.33	4266666.67	880033.33	N/D
E9PS65	1466800.00	8200000.00	N/D	2666666.67	5720000.00	4533333.33	11646666.67	5900000.00
F2Z2W8	N/D	N/D	N/D	N/D	N/D	N/D	2000000.00	1333.33	N/D
F2Z393	23666666.67	5100000.00	4400000.00	1533356.67	4233333.33	16000000.00	6933333.33	16000000.00
F2Z3J9	N/D	4900000.00	7600000.00	3866666.67	3600000.00	9800000.00	834000.00	N/D
F5GWW2	N/D	1466666.67	N/D	N/D	N/D	4966666.67	1833333.33	5900000.00
F5GX11	N/D	470000.00	8533333.33	4300000.00	2233333.33	400.00	3233333.33	14000000.00
F5GXC7	N/D	1466666.67	N/D	N/D	N/D	4966666.67	1833333.33	5900000.00
F5GXK7	20600000.00	17333333.33	60000000.00	23466666.67	34666666.67	27266666.67	26333333.33	10000.00
F5GXY0	N/D	N/D	4200000.00	N/D	N/D	980000.00	2733333.33	N/D
F5GXY2	3666666.67	6766666.67	10733333.33	5133666.67	4300000.00	13100000.00	5566666.67	60000000.00
F5GYU2	3666666.67	6766666.67	10733333.33	5133666.67	4300000.00	13100000.00	5566666.67	60000000.00
F5GYU3	20600000.00	17333333.33	60000000.00	23466666.67	34666666.67	27266666.67	26333333.33	10000.00
F5GYZ5	N/D	4000000.00	10400000.00	8133333.33	6933333.33	14666666.67	5033333.33	28000000.00
F5GZI0	N/D	N/D	N/D	N/D	590000.00	N/D	5000000.00	N/D
F5GZQ4	N/D	1466666.67	1600000.00	N/D	N/D	4966666.67	1566666.67	5900000.00
F5H037	N/D	2400000.00	1600000.00	N/D	N/D	3000000.00	2133333.33	N/D
F5H0C8	N/D	70.00	N/D	N/D	2666666.67	2400000.00	2833333.33	1400000.00
F5H265	20600000.00	17333333.33	60000000.00	23466666.67	34666666.67	27266666.67	26333333.33	10000.00
F5H2D2	N/D	N/D	N/D	N/D	N/D	3466666.67	N/D	12000000.00
F5H388	20600000.00	17333333.33	60000000.00	23466666.67	34666666.67	27266666.67	26333333.33	10000.00
F5H3C5	N/D	4000000.00	10400000.00	8133333.33	6933333.33	14666666.67	5033333.33	28000000.00
F5H3X6	N/D	N/D	1200000.00	N/D	930000.00	3200000.00	3233333.33	600.00
F5H5J4	N/D	1466666.67	1600000.00	N/D	3266666.67	4966666.67	1566666.67	5900000.00
F5H6W8	N/D	1466666.67	1600000.00	N/D	3266666.67	4966666.67	1566666.67	5900000.00
F5H747	20600000.00	17333333.33	60000000.00	23466666.67	34666666.67	27266666.67	26333333.33	10000.00
F5H793	2433333.33	5466666.67	9000000.00	5966666.67	4200000.00	6300000.00	4166666.67	44000000.00
F5H7S3	N/D	N/D	N/D	N/D	3266666.67	N/D	N/D	N/D
F5H8H6	N/D	1466666.67	N/D	N/D	N/D	4966666.67	1833333.33	5900000.00
F6UJC5	N/D	N/D	N/D	N/D	2966666.67	N/D	N/D	N/D
F6UXX1	N/D	N/D	1533333.33	N/D	8266666.67	4866666.67	3033333.33	N/D
F8VPA1	N/D	N/D	N/D	N/D	3700000.00	4566666.67	4566666.67	6666666.67
F8VQ14	N/D	N/D	N/D	1300000.00	220033.33	1466666.67	N/D	4500000.00
F8VRP1	N/D	N/D	N/D	N/D	3700000.00	6455576.00	6666666.67	6666666.67
F8VW21	N/D	720000.00	N/D	866666.67	330000.00	2900000.00	723333.33	3600000.00
F8VY02	N/D	N/D	N/D	1400000.00	1300000.00	1700000.00	N/D	13000000.00
F8VYE8	N/D	1666666.67	1800000.00	833533.33	1086666.67	3600000.00	2133333.33	N/D
F8W079	N/D	2766666.67	1500266.67	5400000.00	26666666.67	3400000.00	100.00	13000000.00
F8W1K8	N/D	N/D	N/D	N/D	N/D	2400000.00	N/D	N/D
F8W1S4	N/D	N/D	N/D	N/D	4566666.67	6666666.67	6666666.67	6666666.67
F8W809	4400000.00	5133333.33	6800000.00	6500000.00	12453333.33	1266666.67	21566666.67	3300000.00
F8W888	2800000.00	2600000.00	N/D	4500000.00	766900.00	2800000.00	2033333.33	2400000.00
F8WCF6	N/D	1700000.00	2600000.00	1666666.67	1500000.00	2700000.00	936666.67	6300000.00
F8WCR4	N/D	N/D	N/D	N/D	N/D	2000000.00	1333.33	N/D
G3V1A4	N/D	2933333.33	2900000.00	6866666.67	2786666.67	1000.00	3266666.67	36000000.00
G3V227	N/D	N/D	2800000.00	1200000.00	2300000.00	100.00	4366666.67	5533333.33
G3V251	N/D	5700000.00	6400066.67	3500000.00	1500066.67	14666666.67	2266733.33	35000000.00
G3V2J8	N/D	N/D	N/D	1200000.00	9500000.00	466.67	6213333.33	6900000.00
G3V2Y4	N/D	N/D	N/D	N/D	3800000.00	N/D	4500000.00	N/D
G3V393	N/D	N/D	N/D	2500000.00	N/D	6200000.00	800000.00	N/D
G3V3R6	N/D	3300000.00	7200000.00	3166666.67	9500000.00	8700000.00	1250000.00	45000000.00
G3V4C1	N/D	5700000.00	6400066.67	3500000.00	1133400.00	14666666.67	2266733.33	35000000.00
G3V4X5	N/D	N/D	N/D	N/D	1666666.67	3466666.67	1566666.67	8200000.00
G3V555	N/D	5700000.00	6400066.67	3500000.00	1500066.67	14666666.67	2266733.33	35000000.00
G3V5L0	N/D	N/D	N/D	N/D	3800000.00	N/D	4233333.33	N/D
G3V5W1	N/D	N/D	2800000.00	1200000.00	2300000.00	100.00	4900000.00	5066666.67
H0Y3Z3	1326666.67	2233333.33	2800000.00	1996666.67	9866666.67	667133.33	8033333.33	1500000.00
H0Y400	N/D	N/D	N/D	N/D	5800000.00	1900000.00	400.00	4666666.67
H0Y4Z6	N/D	N/D	N/D	N/D	3366666.67	466.67	7500000.00	380000.00
H0Y6E7	N/D	N/D	N/D	1500000.00	9286666.67	2800000.00	N/D	4000000.00

H0Y7A7	N/D	N/D	N/D	N/D	N/D	N/D	1933333.33	N/D
H0Y8L3	N/D	N/D	N/D	N/D	3333333.33	1400000.00	2333333.33	N/D
H0YA96	N/D	3600000.00	N/D	600200.00	900.00	3000000.00	4266666.67	840000.00
H0YAB0	N/D	N/D	N/D	1300000.00	7566666.67	N/D	3000033.33	5300000.00
H0YAK4	N/D	N/D	N/D	N/D	2966666.67	1700000.00	233.33	N/D
H0YAR2	N/D	N/D	N/D	N/D	6500000.00	N/D	500.00	N/D
H0YB22	N/D	N/D	N/D	1900000.00	N/D	N/D	570000.00	N/D
H0YB80	N/D	1300000.00	N/D	2500000.00	3300000.00	2800000.00	80.00	13000000.00
H0YB86	N/D	N/D	N/D	N/D	6500000.00	N/D	500.00	N/D
H0YCY8	N/D	N/D	N/D	N/D	3366666.67	2400000.00	N/D	4800000.00
H0YD14	N/D	N/D	N/D	N/D	1666666.67	1466666.67	1833333.33	N/D
H0YDG2	2200000.00	N/D	N/D	N/D	N/D	N/D	N/D	N/D
H0YDZ7	N/D	2800000.00	N/D	N/D	5400000.00	8000000.00	7566666.67	800.00
H0YEP8	N/D	1200000.00	N/D	N/D	1300000.00	N/D	2133333.33	N/D
H0YEU2	N/D	N/D	N/D	N/D	1300000.00	N/D	450000.00	7333333.33
H0YF32	N/D	N/D	N/D	N/D	1266666.67	N/D	450000.00	N/D
H0YFX9	N/D	1400000.00	N/D	5800000.00	8900000.00	N/D	1733333.33	56666666.67
H0YH81	N/D	4100000.00	2533600.00	5900000.00	33333333.33	4400000.00	13333466.67	13000000.00
H0YI37	N/D	N/D	N/D	N/D	2333666.67	2300000.00	533733.33	2666666.67
H0YKF0	N/D	N/D	N/D	N/D	590000.00	1800000.00	N/D	1200000.00
H0YKN4	N/D	780000.00	70.00	N/D	20000000.00	100.00	900000.00	1926666.67
H0YKP3	N/D	N/D	N/D	N/D	3266666.67	N/D	N/D	N/D
H0YKS0	N/D	N/D	7533333.33	8800000.00	N/D	7800000.00	N/D	8900000.00
H0YL80	N/D	N/D	N/D	N/D	3266666.67	N/D	N/D	N/D
H0YLV6	N/D	2633333.33	2966666.67	4733333.33	3633333.33	8400000.00	4100000.00	15000000.00
H0YMA1	N/D	3800000.00	4533333.33	3000000.00	5366666.67	9200000.00	1833333.33	26000000.00
H0YNB8	N/D	N/D	N/D	2666666.67	2200000.00	400.00	5000000.00	4566666.67
H3BMQ8	8366666.67	9666766.67	5766766.67	5466666.67	11000060.00	15233333.33	17233433.33	15333333.33
H3BND3	N/D	N/D	N/D	N/D	1333333.33	1800000.00	N/D	400.00
H3BNV2	N/D	9400000.00	5933333.33	8800000.00	1186666.67	13000000.00	8233333.33	42000000.00
H3BP20	N/D	1300000.00	N/D	1500000.00	266.67	N/D	N/D	N/D
H3BPK3	N/D	N/D	N/D	1200000.00	2353333.33	2300000.00	N/D	400.00
H3BPQ4	N/D	N/D	N/D	1200000.00	2353333.33	2300000.00	N/D	400.00
H3BQ34	11466666.67	6066666.67	2733433.33	9066666.67	49100000.00	31000000.00	41266800.00	22000000.00
H3BQB1	N/D	N/D	N/D	N/D	686666.67	1400000.00	N/D	2400000.00
H3BSC1	N/D	N/D	N/D	N/D	100.00	N/D	N/D	3500000.00
H3BSW3	N/D	N/D	N/D	N/D	80.00	1800000.00	N/D	N/D
H3BTN5	12866666.67	6300000.00	4266666.67	8433333.33	51533433.33	35000000.00	43166666.67	3000.00
H7BY16	N/D	2300000.00	1533333.33	3400000.00	20200000.00	4700000.00	13766666.67	16000000.00
H7BYY1	N/D	N/D	N/D	N/D	3266666.67	N/D	N/D	N/D
H7BZ94	2933333.33	1800066.67	3126666.67	1230133.33	13543333.33	6866666.67	10666666.67	1500000.00
H7C0A3	N/D	N/D	4200000.00	1400000.00	1500000.00	2466666.67	N/D	666.67
H7C1H2	N/D	N/D	N/D	N/D	3666666.67	3600000.00	2333333.33	N/D
H7C1V0	N/D	2900000.00	N/D	2500000.00	4466666.67	7300000.00	3133333.33	13000000.00
H7C2V1	N/D	N/D	N/D	N/D	2933333.33	N/D	1733333.33	N/D
H7C3Z9	N/D	N/D	2800000.00	2500000.00	N/D	4400000.00	1733333.33	2000.00
H7C4C7	20600000.00	17333333.33	16000000.00	23466666.67	24666666.67	27266666.67	26333333.33	10000000.00
H7C5W8	N/D	N/D	N/D	N/D	366.67	N/D	2010000.00	N/D
I3L1U9	18000000.00	23333333.33	7033333.33	28000333.33	113043333.33	55333333.33	82300000.00	36666666.67
I3L397	N/D	2500000.00	N/D	4066666.67	1900000.00	8700000.00	1633333.33	5900000.00
I3L398	2933333.33	1800066.67	5066666.67	1230133.33	13543333.33	6866666.67	10666666.67	1500000.00
I3L3U9	N/D	N/D	N/D	N/D	503333.33	1400000.00	N/D	3666666.67
I3L4N8	13333666.67	22400000.00	5700000.00	17667000.00	87366666.67	55333666.67	58966666.67	39000000.00
I3L504	N/D	2500000.00	N/D	4066666.67	1900000.00	8700000.00	1633333.33	5900000.00
J3KN67	N/D	N/D	N/D	N/D	3033333.33	N/D	N/D	N/D
J3KPD9	400466.67	5200066.67	11800000.00	6233366.67	13666666.67	18400000.00	4166666.67	40000000.00
J3KPE3	2000000.00	2400000.00	N/D	516666.67	1286666.67	3300000.00	2733333.33	4400000.00
J3KPS3	19333333.33	11633333.33	13300000.00	12033333.33	2203333.33	63666666.67	4736666.67	24333333.33
J3KQ32	N/D	N/D	N/D	N/D	1900000.00	2300000.00	1566666.67	N/D
J3KRD5	N/D	N/D	N/D	N/D	N/D	3966666.67	1700000.00	4266666.67
J3KRE2	N/D	N/D	N/D	1300000.00	1366666.67	3800000.00	N/D	N/D
J3KS13	N/D	N/D	N/D	N/D	N/D	N/D	4366666.67	N/D
J3KSV5	N/D	N/D	N/D	N/D	N/D	3966666.67	1700000.00	4266666.67
J3KTF8	N/D	N/D	N/D	1300000.00	1366666.67	3800000.00	N/D	N/D
J3KTM9	N/D	N/D	N/D	N/D	3800000.00	1700000.00	4466666.67	N/D
J3KTN0	N/D	2733333.33	2600000.00	N/D	626666.67	2600000.00	3466666.67	N/D
J3QKK1	N/D	N/D	N/D	N/D	3166666.67	N/D	133.33	N/D
J3QKN0	20600000.00	17333333.33	60000000.00	23466666.67	34666666.67	27266666.67	26333333.33	10000.00
J3QKT2	N/D	N/D	N/D	N/D	3966666.67	1700000.00	3033333.33	N/D
J3QLI9	3533333.33	3300033.33	4300000.00	200.00	3200000.00	6400000.00	2500000.00	4600000.00
J3QLU1	N/D	N/D	N/D	N/D	N/D	3966666.67	1700000.00	4266666.67
J3QQX2	N/D	N/D	N/D	1300000.00	1366666.67	3800000.00	N/D	N/D
J3QR27	N/D	N/D	N/D	N/D	N/D	3966666.67	1700000.00	4266666.67
J3QR64	N/D	2733333.33	2600000.00	N/D	3300000.00	2466666.67	3466666.67	N/D
J3QS69	N/D	2733333.33	2600000.00	N/D	1356666.67	2466666.67	3466666.67	N/D
J3QSA3	17733333.33	5933333.33	38733333.33	8033333.33	19000000.00	17066666.67	21800000.00	6666.67
J3QSV6	N/D	N/D	N/D	N/D	503333.33	1400000.00	N/D	3666666.67
K7EJH8	N/D	N/D	N/D	N/D	N/D	N/D	2033333.33	N/D
K7EJP1	N/D	1200000.00	N/D	N/D	N/D	N/D	6366666.67	N/D
K7EK07	166.67	10166666.67	10600000.00	6333333.33	600.00	800.00	1666766.67	94000000.00
K7EKH5	7266666.67	5633333.33	366.67	1666666.67	8433333.33	12333333.33	10166666.67	24000000.00
K7ELL7	N/D	N/D	N/D	N/D	3566666.67	N/D	N/D	N/D
K7ELN9	N/D	1113333.33	4533333.33	600.00	333666.67	8900000.00	1667133.33	3600000.00
K7ELR7	3533333.33	576690.00	900.00	5800000.00	31666666.67	6333333.33	5666700.00	9000000.00
K7ELW0	N/D	2466666.67	2533333.33	2200000.00	N/D	N/D	2833333.33	N/D
K7ELW5	N/D	1466666.67	2600000.00	N/D	N/D	4666666.67	1833333.33	5200000.00

K7EMH1	N/D	N/D	N/D	2300000.00	1435333.33	2700000.00	N/D	3666666.67
K7EMN2	2200000.00	2400000.00	3200000.00	4000000.00	12000043.33	6400000.00	19200000.00	6666666.67
K7EMV3	166.67	10166666.67	10600000.00	6333333.33	600.00	800.00	1666766.67	94000000.00
K7ENJ4	N/D	3466666.67	2700000.00	N/D	8800000.00	4200000.00	3900000.00	5600000.00
K7ENT6	N/D	N/D	N/D	N/D	3266666.67	N/D	N/D	N/D
K7EP68	N/D	N/D	N/D	N/D	3266666.67	N/D	N/D	N/D
K7EPF6	1866666.67	1600000.00	3200000.00	N/D	8333376.67	3400000.00	9866666.67	5900000.00
K7EPJ3	N/D	980000.00	1533333.33	1500000.00	2933333.33	2200000.00	3566666.67	1900000.00
K7ERG3	N/D	N/D	N/D	N/D	3266666.67	N/D	N/D	N/D
K7ERK8	2466666.67	496696.67	9300000.00	2666666.67	18000000.00	4133333.33	2966700.00	N/D
K7ES00	166.67	10166666.67	10600000.00	6333333.33	600.00	800.00	1666766.67	94000000.00
L8B4R0	20600000.00	17333333.33	60000000.00	23466666.67	34666666.67	27266666.67	26333333.33	10000.00
L8B4Z6	20600000.00	17333333.33	60000000.00	23466666.67	34666666.67	27266666.67	26333333.33	10000.00
M0R117	N/D	3000000.00	6300000.00	3500000.00	1366666.67	2466666.67	600.00	3800000.00
M0R1M6	3933333.33	16000000.00	32667333.33	21300000.00	29666666.67	17766666.67	27600000.00	69000000.00
M0R3D6	N/D	3000000.00	6300000.00	3500000.00	1366666.67	2466666.67	600.00	3800000.00
O14992	N/D	N/D	6800000.00	100.00	6966666.67	6800000.00	400.00	N/D
O15144	N/D	N/D	N/D	2400000.00	3600000.00	1200000.00	N/D	133.33
O43175	N/D	N/D	N/D	N/D	3383333.33	1200000.00	4033333.33	2500000.00
O43242	N/D	N/D	N/D	N/D	1366666.67	1466666.67	2033333.33	N/D
O43390	N/D	N/D	N/D	N/D	4033333.33	N/D	3133333.33	N/D
O43707	N/D	N/D	N/D	N/D	3300000.00	3200000.00	3066700.00	N/D
O43776	N/D	N/D	100.00	N/D	2966666.67	300.00	233.33	N/D
O60812	N/D	6366666.67	200.00	3200000.00	3366666.67	16000000.00	2430000.00	18000000.00
O60814	N/D	4400000.00	N/D	5600000.00	3366666.67	2466666.67	4400000.00	16000000.00
O75083	N/D	N/D	N/D	N/D	N/D	2300000.00	1833333.33	N/D
O75369	N/D	N/D	N/D	N/D	3073333.33	N/D	6500000.00	2666666.67
O75390	N/D	2000000.00	1700000.00	N/D	4553333.33	5066666.67	3453333.33	N/D
O75526	N/D	N/D	N/D	1500000.00	1300000.00	N/D	N/D	4000000.00
O76003	2300000.00	N/D	N/D	N/D	N/D	2433333.33	8233333.33	N/D
P00505	2100000.00	8900000.00	4600000.00	8600000.00	5906666.67	9166666.67	7000000.00	2600000.00
P00558	9100000.00	14533333.33	12300000.00	9333333.33	1806766.67	54633333.33	53433333.33	50000000.00
P01023	N/D	1800000.00	4700000.00	3800000.00	N/D	2700000.00	3233333.33	N/D
P01834	N/D	N/D	9800000.00	5200000.00	3300000.00	466.67	N/D	16000000.00
P01857	2600000.00	2300000.00	N/D	4400000.00	12666666.67	4200000.00	5066666.67	N/D
P01859	N/D	N/D	N/D	N/D	500.00	2300000.00	3033333.33	N/D
P01871	N/D	N/D	N/D	N/D	4333333.33	N/D	3666666.67	N/D
P02765	9800000.00	2400000.00	3580000.00	243766.67	3966666.67	4733333.33	2833333.33	N/D
P02792	333.33	1800000.00	1300000.00	2300000.00	2766666.67	4700000.00	567066.67	200.00
P04075	19333333.33	11633333.33	13300000.00	12033333.33	2203333.33	63666666.67	4736666.67	24333333.33
P04083	N/D	1333333.33	1700000.00	1533333.33	2533333.33	3600000.00	733400.00	15000000.00
P04179	N/D	4200000.00	10166666.67	7100000.00	6933333.33	19000000.00	5300000.00	28000000.00
P04220	N/D	N/D	N/D	N/D	4333333.33	N/D	3666666.67	N/D
P04406	28666666.67	32333666.67	20466666.67	70166666.67	85633333.33	190000000.00	41866666.67	174100000.00
P04843	N/D	N/D	N/D	N/D	1666666.67	1900000.00	3566666.67	N/D
P05091	2100000.00	N/D	100.00	1500000.00	626666.67	2366666.67	3233333.33	N/D
P05120	3666666.67	2033333.33	9900000.00	N/D	13666666.67	12000000.00	3733333.33	13000000.00
P05455	N/D	N/D	800000.00	N/D	3566666.67	2200000.00	133.33	N/D
P06733	41333333.33	16466666.67	13766666.67	3366666.67	112600000.00	60000000.00	4340000.00	23833333.33
P06744	4400000.00	1666690.00	5066666.67	1487000.00	15460000.00	13866666.67	1334666.67	4133333.33
P06865	N/D	1300000.00	N/D	1500000.00	266.67	N/D	N/D	N/D
P07205	2000266.67	800.00	2533333.33	6000000.00	25566666.67	5333600.00	41933333.33	34000000.00
P07737	2566666.67	4266666.67	7533333.33	2833333.33	3600000.00	13966666.67	5166666.67	25000000.00
P07741	N/D	N/D	N/D	N/D	1666666.67	1400000.00	N/D	2400000.00
P07900	3933333.33	2800000.00	5866666.67	1900000.00	26666666.67	7733333.33	7033333.33	19000000.00
P07910	N/D	5700000.00	6400066.67	3500000.00	1133400.00	14666666.67	2266733.33	35000000.00
P07951	N/D	N/D	N/D	N/D	3266666.67	N/D	N/D	N/D
P08174	2933333.33	1133400.00	N/D	N/D	12666666.67	2700000.00	2233333.33	N/D
P08238	2433333.33	2166666.67	3400000.00	2233333.33	13666666.67	6966666.67	4266666.67	15000000.00
P09382	166.67	1633333.33	1000.00	1500030.00	N/D	400.00	166.67	7466666.67
P09622	2366666.67	2000000.00	N/D	2500000.00	393433.33	3366666.67	3866666.67	1200000.00
P09661	N/D	N/D	N/D	N/D	2666666.67	1400000.00	N/D	N/D
P09936	N/D	2000000.00	1533333.33	2133333.33	8900000.00	7400000.00	N/D	8666666.67
P09960	N/D	N/D	N/D	N/D	3666666.67	N/D	2733333.33	N/D
P0C0S8	N/D	1400000.00	N/D	5800000.00	8900000.00	N/D	1733333.33	56666666.67
P0DMV8	2800030.00	1500030.00	6400066.67	3266666.67	2366666.67	1800030.00	3266666.67	1500030.00
P0DMV9	2866666.67	4700000.00	6233333.33	1773333.33	9403333.33	6266666.67	11566666.67	N/D
P10155	N/D	630000.00	N/D	N/D	3266666.67	100.00	3333333.33	N/D
P10599	3266666.67	1433333.33	N/D	2000100.00	3466666.67	4000000.00	1746700.00	N/D
P11216	2366666.67	N/D	N/D	N/D	8600000.00	4700000.00	15666666.67	850000.00
P11279	2600000.00	1800000.00	840000.00	N/D	3900000.00	200.00	6466666.67	N/D
P11413	1966933.33	6000000.00	3400000.00	800266.67	23400000.00	19966666.67	37000000.00	1000.00
P12081	N/D	N/D	N/D	N/D	3900000.00	N/D	4800000.00	N/D
P12955	N/D	N/D	N/D	N/D	4266666.67	1700000.00	2933333.33	N/D
P13010	2100000.00	1466666.67	3400000.00	200.00	6933333.33	4900000.00	1166666.67	4500000.00
P13473	6700000.00	2533333.33	2300000.00	6500000.00	9900000.00	8366666.67	3700000.00	N/D
P13489	N/D	N/D	N/D	N/D	3666666.67	1400000.00	3933333.33	N/D
P13639	2266666.67	1733333.33	N/D	2013333.33	12200000.00	6000000.00	12066666.67	5900000.00
P13667	N/D	N/D	N/D	N/D	2853333.33	N/D	3733333.33	1800000.00
P13804	N/D	N/D	N/D	N/D	590000.00	1800000.00	N/D	1200000.00
P14174	2000000.00	1166666.67	28733333.33	7333333.33	3266666.67	2000.00	3766666.67	85000000.00
P14314	N/D	N/D	N/D	N/D	3566666.67	N/D	N/D	N/D
P14678	N/D	2300000.00	7200000.00	1900000.00	2933333.33	3000000.00	233.33	6866666.67
P14923	2066666.67	N/D	N/D	N/D	716666.67	1400000.00	2233333.33	N/D
P15121	N/D	4466666.67	1866933.33	2666666.67	N/D	7300000.00	3666666.67	6666666.67
P15311	N/D	N/D	N/D	N/D	6000000.00	5133333.33	600.00	N/D

P15531	400466.67	667066.67	11800000.00	6433366.67	13666666.67	16400000.00	2800100.00	40000000.00
P15586	2366666.67	1010000.00	N/D	3200000.00	5000000.00	2766666.67	1053433.33	N/D
P16083	N/D	N/D	N/D	580000.00	293333.33	990000.00	N/D	2400000.00
P16152	N/D	N/D	N/D	N/D	266.67	200.00	N/D	1000.00
P17066	1733400.00	4700000.00	N/D	1306666.67	8313333.33	2866733.33	12733333.33	N/D
P17174	N/D	3000000.00	1600000.00	N/D	7266666.67	2466666.67	3333353.33	N/D
P17931	1866666.67	1730000.00	9300000.00	4633333.33	12666666.67	400666.67	1216666.67	50000000.00
P18124	N/D	480000.00	N/D	N/D	780000.00	1800000.00	840000.00	3200000.00
P19367	N/D	N/D	N/D	N/D	1900000.00	100.00	2233333.33	N/D
P20618	N/D	840000.00	N/D	1160000.00	1800000.00	1700000.00	923333.33	9600000.00
P20671	N/D	1400000.00	N/D	5800000.00	8900000.00	N/D	1733333.33	56666666.67
P21266	N/D	660000.00	3000000.00	N/D	N/D	3800000.00	1566666.67	5000000.00
P21333	166.67	N/D	N/D	N/D	3266666.67	166.67	4233333.33	N/D
P21796	N/D	1466666.67	4700000.00	N/D	1300000.00	4200000.00	2233333.33	1000.00
P22392	400466.67	5200066.67	11800000.00	6433366.67	13666666.67	18400000.00	4166666.67	40000000.00
P22626	N/D	4100000.00	1933333.33	600466.67	1766666.67	5066666.67	1566666.67	9200000.00
P23246	N/D	N/D	N/D	2500000.00	5000000.00	4900000.00	880000.00	2000000.00
P23381	2266666.67	1700000.00	1440000.00	1460000.00	2344333.33	6733333.33	19000000.00	4500000.00
P23396	N/D	N/D	N/D	N/D	1266666.67	N/D	450000.00	N/D
P23526	2966666.67	1800000.00	7200000.00	2600000.00	800.00	1166966.67	6200000.00	840000.00
P23528	N/D	2933333.33	2900000.00	6866666.67	2786666.67	1000.00	2933333.33	36000000.00
P25786	N/D	470000.00	6900000.00	4300000.00	1066800.00	400.00	3233333.33	14000000.00
P25788	N/D	1466666.67	N/D	1600053.33	886666.67	4400000.00	1566666.67	8666666.67
P25789	N/D	2900000.00	3533333.33	2600000.00	8900000.00	N/D	2033333.33	18000000.00
P26038	N/D	N/D	886666.67	4200000.00	6433333.33	7300000.00	8600000.00	1600000.00
P26447	N/D	1300000.00	3900000.00	N/D	3333333.33	6000000.00	1666666.67	14000000.00
P26599	N/D	1800000.00	2600000.00	N/D	3300000.00	6200000.00	2133333.33	3200000.00
P26639	N/D	580000.00	N/D	1400000.00	3966666.67	2200000.00	4600000.00	N/D
P26641	N/D	770000.00	4200000.00	1766700.00	3166666.67	5466666.67	1900000.00	8000000.00
P27348	N/D	3300000.00	2300000.00	3666666.67	703333.33	6400000.00	960000.00	N/D
P27635	N/D	N/D	N/D	1666666.67	100.00	N/D	N/D	5000000.00
P27824	N/D	N/D	N/D	N/D	7266666.67	4200000.00	600.00	N/D
P28070	N/D	2466666.67	2533333.33	2200000.00	N/D	N/D	2833333.33	N/D
P28072	N/D	946666.67	N/D	1733333.33	3666666.67	3466666.67	950000.00	6800000.00
P28074	N/D	N/D	N/D	1000000.00	2933333.33	2400000.00	2133333.33	5400000.00
P29401	5800000.00	18000000.00	8300000.00	11500066.67	2934333.33	9600000.00	13500000.00	12240000.00
P30040	N/D	1466666.67	N/D	1400000.00	1300000.00	2000000.00	1833333.33	16666666.67
P30043	N/D	N/D	N/D	N/D	N/D	1200000.00	N/D	N/D
P30048	N/D	2666666.67	2553333.33	3600000.00	4333333.33	1000.00	1566666.67	1000.00
P30101	2100000.00	200.00	1200000.00	1200000.00	2001333.33	5166666.67	30433333.33	600.00
P30153	N/D	N/D	N/D	N/D	266.67	1700000.00	4400000.00	N/D
P32929	N/D	1600000.00	N/D	N/D	3800000.00	100.00	333.33	N/D
P34932	N/D	N/D	6800000.00	100.00	6966666.67	6800000.00	400.00	N/D
P35268	N/D	N/D	N/D	2666666.67	2200000.00	400.00	5000000.00	4566666.67
P35580	N/D	N/D	800000.00	N/D	3566666.67	8900000.00	4000000.00	N/D
P35613	2533333.33	1300000.00	N/D	2666666.67	6666666.67	3600000.00	3720000.00	490000.00
P36873	N/D	1666666.67	1800000.00	833533.33	1086666.67	3600000.00	2133333.33	N/D
P37837	18533333.33	1900400.00	2000400.00	2400023.33	4666666.67	12533333.33	5933333.33	22000000.00
P38159	N/D	N/D	N/D	1500000.00	4053333.33	2800000.00	N/D	4000000.00
P38646	1866666.67	1800000.00	N/D	2500000.00	8000000.00	400300.00	10963333.33	3200000.00
P39656	N/D	N/D	N/D	N/D	2966666.67	2000000.00	3333333.33	N/D
P39687	N/D	970000.00	N/D	1800000.00	1000000.00	3200000.00	970000.00	3200000.00
P40925	N/D	4800000.00	N/D	4666666.67	N/D	4466666.67	4800000.00	N/D
P40926	2233333.33	7400000.00	17000000.00	8200000.00	5066666.67	24500000.00	3900000.00	19430000.00
P41091	N/D	N/D	N/D	1500000.00	N/D	4700000.00	8333333.33	3366666.67
P41250	N/D	1700000.00	N/D	N/D	867000.00	400.00	5500000.00	N/D
P41252	N/D	N/D	N/D	N/D	3366666.67	2900000.00	2733333.33	N/D
P42330	N/D	2000000.00	N/D	400.00	1986666.67	6700000.00	4500000.00	15000000.00
P46781	N/D	2300000.00	4300000.00	6300000.00	5433333.33	3000000.00	2933400.00	4400000.00
P48163	N/D	N/D	N/D	N/D	500.00	930000.00	3933333.33	N/D
P48594	N/D	N/D	N/D	N/D	N/D	1700000.00	N/D	N/D
P48740	1870000.00	2133333.33	1270000.00	2466666.67	1666666.67	1290666.67	1433333.33	2466666.67
P49411	N/D	N/D	N/D	N/D	5600000.00	2400000.00	1463333.33	N/D
P49591	N/D	N/D	N/D	N/D	3333333.33	N/D	1833333.33	N/D
P49720	870000.00	2133333.33	700.00	2466666.67	1666666.67	290666.67	4033333.33	N/D
P49721	N/D	2800033.33	4333333.33	1223600.00	866766.67	9000000.00	4400000.00	N/D
P49748	N/D	N/D	N/D	N/D	3300000.00	3200000.00	N/D	N/D
P49773	N/D	N/D	N/D	1300000.00	293333.33	300.00	N/D	4500000.00
P50454	N/D	1200000.00	N/D	N/D	2933333.33	N/D	2833333.33	N/D
P50502	N/D	N/D	N/D	N/D	3266666.67	N/D	N/D	N/D
P50991	N/D	N/D	1900000.00	2000000.00	3033333.33	300.00	1120000.00	N/D
P51148	N/D	633333.33	2400000.00	N/D	860000.00	4300000.00	3333333.33	6300000.00
P51149	N/D	1400000.00	2300000.00	1400000.00	1500000.00	3400000.00	N/D	4800000.00
P51858	N/D	N/D	100.00	N/D	N/D	N/D	N/D	6000000.00
P52209	2066666.67	3446666.67	3800133.33	15000000.00	1553.33	601333.33	23466666.67	5900000.00
P52895	N/D	N/D	2700000.00	2500000.00	696666.67	5366666.67	N/D	13000000.00
P53004	N/D	N/D	N/D	2200000.00	3266666.67	2900000.00	3733333.33	2200000.00
P53396	N/D	N/D	N/D	1200000.00	N/D	2800000.00	4033333.33	2233333.33
P53634	N/D	N/D	N/D	N/D	3366666.67	2400000.00	N/D	4800000.00
P53675	N/D	N/D	N/D	N/D	3266666.67	920000.00	246900.00	N/D
P55145	N/D	100.00	N/D	1400000.00	N/D	N/D	80.00	3800000.00
P55786	1333333.33	1600000.00	N/D	1400000.00	600000.00	2266666.67	6400000.00	N/D
P56537	N/D	N/D	N/D	N/D	1266666.67	4200000.00	N/D	666.67
P57053	N/D	4400000.00	N/D	5600000.00	3366666.67	2466666.67	4400000.00	16000000.00
P59998	N/D	1700000.00	2600000.00	1666666.67	1500000.00	2700000.00	933333.33	6300000.00
P60174	N/D	2133466.67	3700000.00	3933333.33	2233366.67	1000.00	6500000.00	20000000.00

P60900	N/D	2200000.00	6033333.33	1666666.67	3600000.00	6000000.00	2833333.33	N/D
P60903	N/D	4300000.00	N/D	3000000.00	7166666.67	6800000.00	4333333.33	N/D
P61204	N/D	N/D	2400000.00	360000.00	1833333.33	9000000.00	1733333.33	3000000.00
P61247	N/D	N/D	N/D	N/D	830000.00	100.00	N/D	4800000.00
P61604	N/D	7800000.00	6400000.00	10933333.33	5566666.67	9800000.00	1666666.67	62000000.00
P61970	N/D	N/D	6133333.33	N/D	1300000.00	300.00	N/D	8300000.00
P61978	N/D	880000.00	2300000.00	2000000.00	N/D	4666666.67	N/D	300000.00
P61981	N/D	4400000.00	990000.00	1926666.67	300.00	3900000.00	2933333.33	18000000.00
P62081	N/D	N/D	N/D	N/D	30.00	1700000.00	N/D	1666666.67
P62136	N/D	1666666.67	1800000.00	833533.33	1086666.67	3600000.00	2133333.33	N/D
P62158	N/D	N/D	N/D	N/D	N/D	N/D	1933333.33	N/D
P62244	N/D	N/D	3266766.67	N/D	1500000.00	4200000.00	933500.00	6666666.67
P62249	N/D	600000.00	1300000.00	1600000.00	N/D	3400000.00	1233333.33	6000000.00
P62263	N/D	N/D	N/D	1900000.00	N/D	N/D	570000.00	N/D
P62277	N/D	2000000.00	2133666.67	9200000.00	5500000.00	4666666.67	6400000.00	9200000.00
P62280	N/D	N/D	N/D	N/D	3566666.67	N/D	N/D	300.00
P62314	3533333.33	3300033.33	4300000.00	200.00	3200000.00	4966666.67	2500000.00	5300000.00
P62318	N/D	2066666.67	N/D	2133333.33	1326833.33	7000000.00	966666.67	300.00
P62424	N/D	N/D	N/D	N/D	1900000.00	N/D	N/D	5900000.00
P62701	N/D	N/D	N/D	N/D	N/D	2200000.00	660000.00	4666666.67
P62807	N/D	4400000.00	N/D	5600000.00	3366666.67	2466666.67	4400000.00	16000000.00
P62888	N/D	2200000.00	2300000.00	N/D	1300000.00	2800000.00	2566666.67	N/D
P62906	N/D	N/D	N/D	2200000.00	980000.00	5266666.67	N/D	4300000.00
P62937	200.00	1167333.33	2000.00	6800000.00	10066666.67	14666733.33	3766666.67	94000000.00
P62979	20600000.00	17333333.33	60000000.00	23466666.67	34666666.67	27266666.67	26333333.33	10000.00
P63104	N/D	2200000.00	1200000.00	2453333.33	4266666.67	600.00	800066.67	14000000.00
P63162	N/D	2300000.00	7200000.00	1900000.00	2933333.33	3000000.00	233.33	6866666.67
P63241	N/D	2500000.00	N/D	4066666.67	1900000.00	8700000.00	1600000.00	5900000.00
P63261	16000333.33	23233333.33	9833333.33	14000333.33	85700000.00	55333666.67	58966666.67	49000000.00
P67936	N/D	N/D	N/D	N/D	3266666.67	N/D	N/D	N/D
P68032	2900000.00	2400000.00	2700000.00	4666666.67	5000.00	968000.00	18333333.33	5900000.00
P68133	2900000.00	2400000.00	2700000.00	4666666.67	5000.00	968000.00	18333333.33	5900000.00
P68431	166.67	10166666.67	10600000.00	6333333.33	600.00	800.00	1633433.33	94000000.00
P83876	2366666.67	980000.00	N/D	N/D	N/D	2166666.67	5500000.00	7166666.67
P84243	166.67	10166666.67	10600000.00	6333333.33	600.00	800.00	1633433.33	94000000.00
P99999	N/D	3466666.67	6133333.33	3800000.00	3166666.67	8600000.00	3033333.33	16666666.67
Q00610	700000.00	N/D	3200000.00	3400000.00	396666.67	3300000.00	5183333.33	N/D
Q01518	N/D	N/D	N/D	N/D	N/D	1400000.00	N/D	N/D
Q02543	N/D	3000000.00	6300000.00	3500000.00	1366666.67	2466666.67	600.00	3800000.00
Q02878	N/D	1466666.67	N/D	2200000.00	N/D	4966666.67	1933333.33	8600000.00
Q04446	N/D	N/D	N/D	N/D	3266666.67	N/D	3133333.33	N/D
Q04837	N/D	1600000.00	6033333.33	2033333.33	1610000.00	4800000.00	3566666.67	4600000.00
Q05639	N/D	N/D	N/D	N/D	6500000.00	400.00	5700000.00	N/D
Q05DH1	N/D	3300000.00	N/D	2066666.67	2533333.33	7800000.00	3666666.67	19000000.00
Q08211	2000000.00	N/D	N/D	N/D	3933333.33	1766666.67	3246666.67	360000.00
Q08AJ6	N/D	970000.00	N/D	1800000.00	6100000.00	3200000.00	970000.00	3200000.00
Q08AJ9	N/D	N/D	N/D	N/D	300.00	N/D	N/D	7166666.67
Q0PHS2	2433333.33	2233333.33	N/D	N/D	10086666.67	8833333.33	18800000.00	4666666.67
Q0PHS3	N/D	N/D	N/D	2666666.67	2200000.00	400.00	5000000.00	4566666.67
Q0VAS5	4666666.67	24200000.00	54433333.33	28600000.00	20200000.00	1772000.00	21333333.33	280866666.67
Q0VGA5	N/D	N/D	N/D	N/D	3333333.33	N/D	1833333.33	N/D
Q12906	N/D	N/D	2200000.00	N/D	1366666.67	4300000.00	3733333.33	3000000.00
Q13011	N/D	N/D	N/D	N/D	936666.67	300.00	N/D	6200000.00
Q13041	N/D	N/D	N/D	N/D	3666666.67	3600000.00	2333333.33	N/D
Q13228	N/D	N/D	N/D	680000.00	3500000.00	1400000.00	1333.33	N/D
Q13724	N/D	N/D	N/D	N/D	300.00	1466666.67	2333333.33	N/D
Q13747	4133333.33	2466666.67	N/D	6600000.00	3566666.67	3533333.33	3366666.67	N/D
Q13838	N/D	980000.00	1533333.33	1500000.00	4200000.00	2000000.00	3100000.00	3300000.00
Q14103	N/D	3600000.00	N/D	600200.00	900.00	3000000.00	3933333.33	840000.00
Q14108	N/D	N/D	N/D	N/D	N/D	N/D	3233333.33	N/D
Q14145	14666666.67	24200000.00	54433333.33	28600000.00	20200000.00	21772000.00	21333333.33	20866666.67
Q14204	N/D	N/D	N/D	N/D	3300000.00	2466666.67	4333333.33	N/D
Q14974	N/D	N/D	N/D	N/D	N/D	2000000.00	4900000.00	4466666.67
Q15019	N/D	N/D	N/D	N/D	416700.00	N/D	N/D	1666666.67
Q15056	N/D	N/D	N/D	N/D	3893333.33	4466666.67	N/D	2600000.00
Q15084	N/D	2000000.00	1633333.33	1413333.33	2533333.33	6400000.00	5833333.33	20000000.00
Q15149	N/D	1300000.00	N/D	N/D	N/D	880000.00	1710000.00	N/D
Q15181	N/D	N/D	N/D	N/D	3566666.67	N/D	N/D	6666666.67
Q15182	N/D	2300000.00	7200000.00	1900000.00	2933333.33	3000000.00	233.33	6866666.67
Q15582	N/D	N/D	N/D	N/D	3333333.33	1400000.00	2333333.33	N/D
Q15631	N/D	N/D	N/D	N/D	980000.00	N/D	N/D	4300000.00
Q15657	N/D	N/D	N/D	N/D	3266666.67	N/D	N/D	N/D
Q16195	1866666.67	N/D	N/D	N/D	N/D	N/D	N/D	N/D
Q16658	313380.00	2200000.00	4900000.00	2666666.67	4986666.67	3443333.33	5066666.67	4300000.00
Q16695	166.67	5733333.33	10700000.00	5700000.00	2600200.00	6600000.00	866833.33	94000000.00
Q16716	10866666.67	6133333.33	2800000.00	2433333.33	2736666.67	18000000.00	2568433.33	39000000.00
Q16768	4366666.67	9600000.00	20000000.00	4966666.67	30000000.00	31233333.33	4600300.00	10000.00
Q16881	4400000.00	5133333.33	6800000.00	6500000.00	13266666.67	1266666.67	21566666.67	3300000.00
Q1ZYL5	N/D	N/D	N/D	N/D	3266666.67	N/D	N/D	N/D
Q2F839	1103000.00	1466666.67	2533333.33	1200000.00	1253000.00	1100000.00	1833333.33	1533300.00
Q2Q9B7	1966933.33	5900000.00	3400000.00	800266.67	22400000.00	19966666.67	36666666.67	1000.00
Q2Q9H2	1966933.33	6000000.00	3400000.00	800266.67	23400000.00	19966666.67	37000000.00	1000.00
Q3BDU5	N/D	N/D	N/D	960000.00	8666666.67	6300000.00	2300000.00	9000000.00
Q53F48	N/D	N/D	N/D	N/D	600000.00	N/D	N/D	1300000.00
Q53F60	N/D	N/D	N/D	N/D	266.67	3000000.00	N/D	1000.00
Q53F64	N/D	1800000.00	N/D	N/D	9200000.00	2300000.00	2133333.33	3066666.67

Q53FB0	N/D	N/D	N/D	1600000.00	1800000.00	2900000.00	N/D	12000000.00
Q53FB6	2100000.00	N/D	100.00	1500000.00	626666.67	2366666.67	3233333.33	N/D
Q53FC7	1733400.00	4700000.00	N/D	1306666.67	8313333.33	2866733.33	12733333.33	N/D
Q53FG3	N/D	N/D	2400000.00	2800000.00	4566666.67	4866666.67	5333333.33	N/D
Q53FT8	N/D	840000.00	N/D	1160000.00	1800000.00	1700000.00	923333.33	9600000.00
Q53FV0	N/D	N/D	N/D	N/D	1266666.67	100.00	N/D	6200000.00
Q53G35	N/D	3766666.67	2800000.00	3273333.33	3633333.33	9400000.00	1566666.67	26000000.00
Q53G58	N/D	N/D	N/D	N/D	3300000.00	N/D	N/D	N/D
Q53G64	N/D	N/D	2800000.00	2500000.00	N/D	4400000.00	1733333.33	16000000.00
Q53G76	16000333.33	23233333.33	9833333.33	14000333.33	85700000.00	55333666.67	58966666.67	49000000.00
Q53G81	N/D	N/D	6533333.33	10533333.33	1986666.67	14000000.00	3360000.00	39000000.00
Q53G83	N/D	N/D	N/D	N/D	1266666.67	N/D	450000.00	7333333.33
Q53G85	N/D	N/D	3700000.00	N/D	13666666.67	4700000.00	3400000.00	9600000.00
Q53G89	N/D	N/D	N/D	N/D	13666666.67	N/D	2400000.00	N/D
Q53GF9	N/D	N/D	N/D	100.00	1800000.00	N/D	N/D	6000000.00
Q53GK6	16000333.33	23233333.33	9833333.33	14000333.33	85700000.00	55333666.67	58966666.67	49000000.00
Q53GN4	N/D	N/D	N/D	N/D	N/D	2300000.00	1833333.33	N/D
Q53GX7	N/D	580000.00	N/D	1400000.00	3966666.67	2200000.00	4600000.00	N/D
Q53GZ6	7433333.33	7800000.00	17966666.67	5000000.00	15386666.67	10966666.67	11333633.33	8300000.00
Q53H17	N/D	N/D	N/D	N/D	N/D	2300000.00	1833333.33	N/D
Q53H26	400.00	N/D	300.00	4666666.67	3566666.67	400.00	N/D	N/D
Q53HA4	N/D	N/D	N/D	N/D	3333333.33	N/D	1833333.33	N/D
Q53HB3	N/D	N/D	N/D	N/D	1800000.00	N/D	N/D	N/D
Q53HC2	N/D	2666666.67	2553333.33	3600000.00	4333333.33	1000.00	1566666.67	1000.00
Q53HD3	N/D	3000000.00	6300000.00	3500000.00	1366666.67	2466666.67	600.00	3800000.00
Q53HE2	N/D	2133466.67	3700000.00	3933333.33	1266766.67	1000.00	6500000.00	20000000.00
Q53HE7	N/D	2300000.00	7200000.00	1900000.00	2933333.33	3000000.00	233.33	6866666.67
Q53HF2	7200000.00	1667200.00	17966666.67	5000000.00	15386666.67	10733333.33	11333633.33	8300000.00
Q53HM9	N/D	N/D	3700000.00	N/D	13666666.67	4700000.00	3400000.00	1000.00
Q53HR2	N/D	N/D	N/D	N/D	3300000.00	3200000.00	N/D	N/D
Q53HR5	N/D	N/D	3700000.00	N/D	13666666.67	4700000.00	3400000.00	1000.00
Q53HV1	N/D	N/D	N/D	N/D	N/D	2200000.00	660000.00	4666666.67
Q53HW2	N/D	800000.00	N/D	866666.67	N/D	3200000.00	800000.00	7066666.67
Q53XL8	N/D	N/D	N/D	N/D	1800000.00	N/D	N/D	N/D
Q549N0	N/D	2900000.00	1300000.00	4000000.00	2620000.00	4600000.00	1833333.33	N/D
Q562L6	22400000.00	27000000.00	6700000.00	14666666.67	149710000.00	38400000.00	90933333.33	66666666.67
Q562R8	18000000.00	23333333.33	7033333.33	28000333.33	113043333.33	55333333.33	82300000.00	53000000.00
Q56A82	16000333.33	23233333.33	19833333.33	14000333.33	15700000.00	15333666.67	15966666.67	19000000.00
Q58FF2	N/D	1400000.00	2400000.00	N/D	3800000.00	2900000.00	333.33	N/D
Q58FF3	N/D	1150000.00	N/D	686666.67	2433333.33	3000000.00	1133433.33	1900000.00
Q59E89	1566666.67	1800000.00	1773333.33	2200000.00	1950000.00	1800000.00	2150000.00	1266666.67
Q59EA2	N/D	N/D	N/D	N/D	3300000.00	N/D	N/D	N/D
Q59EG8	N/D	N/D	N/D	N/D	3666666.67	3600000.00	2333333.33	N/D
Q59EI4	N/D	N/D	N/D	N/D	1366666.67	N/D	N/D	N/D
Q59EI9	N/D	833466.67	933366.67	1966800.00	3600000.00	14000000.00	3833333.33	7333333.33
Q59EJ0	2266666.67	620000.00	100.00	1500000.00	12666666.67	3466666.67	4133333.33	N/D
Q59EJ3	2866666.67	4700000.00	6233333.33	1773333.33	9403333.33	6266666.67	11566666.67	N/D
Q59EJ5	N/D	660000.00	3000000.00	N/D	N/D	3800000.00	1566666.67	5000000.00
Q59EM9	20600000.00	17333333.33	60000000.00	23466666.67	32666666.67	27266666.67	26333333.33	10000.00
Q59EN2	N/D	N/D	N/D	580000.00	366.67	990000.00	N/D	2400000.00
Q59ER5	N/D	N/D	N/D	N/D	N/D	2300000.00	1833333.33	N/D
Q59F85	2466666.67	496696.67	9300000.00	N/D	19000000.00	5066666.67	3200033.33	N/D
Q59FD4	N/D	N/D	N/D	N/D	1900000.00	100.00	2233333.33	N/D
Q59FJ0	N/D	N/D	N/D	3200000.00	N/D	1900000.00	N/D	N/D
Q59G24	N/D	N/D	N/D	866666.67	650000.00	4000000.00	1933333.33	1800000.00
Q59G75	N/D	N/D	N/D	N/D	3366666.67	2900000.00	2733333.33	N/D
Q59GF8	N/D	N/D	6800000.00	100.00	N/D	6800000.00	400.00	6966666.67
Q59GM9	2366666.67	N/D	N/D	N/D	8600000.00	4700000.00	15666666.67	850000.00
Q59GP5	N/D	N/D	N/D	N/D	6500000.00	400.00	5700000.00	N/D
Q59H49	N/D	1466666.67	2600000.00	N/D	N/D	4700000.00	1833333.33	5200000.00
Q5BJF5	N/D	N/D	N/D	N/D	3566666.67	N/D	4000000.00	N/D
Q5CAQ5	N/D	3066666.67	4733333.33	1666666.67	31666666.67	14000000.00	7033333.33	12000000.00
Q5EFE5	2600000.00	2300000.00	N/D	4400000.00	12666666.67	4200000.00	5066666.67	N/D
Q5EK51	4600000.00	4433333.33	6800000.00	277333.33	600.00	7233333.33	4766666.67	N/D
Q5HY54	166.67	N/D	N/D	N/D	3966666.67	166.67	4233333.33	N/D
Q5HYG8	N/D	N/D	N/D	N/D	3566666.67	N/D	4000000.00	N/D
Q5I6Y5	N/D	200.00	N/D	2366666.67	8666666.67	N/D	1866700.00	13000000.00
Q5JYR7	N/D	N/D	N/D	N/D	N/D	3700000.00	1553333.33	N/D
Q5M7Z9	N/D	580000.00	N/D	1400000.00	3966666.67	2200000.00	4600000.00	N/D
Q5PY61	20600000.00	17333333.33	60000000.00	23466666.67	34666666.67	27266666.67	26333333.33	10000.00
Q5QNW6	N/D	4400000.00	N/D	5600000.00	3366666.67	2466666.67	4400000.00	16000000.00
Q5R206	2666666.67	2600000.00	2533333.33	2800000.00	4340000.00	6666666.67	9466666.67	1600000.00
Q5R208	2666666.67	2600000.00	2533333.33	2800000.00	4340000.00	6666666.67	9466666.67	1600000.00
Q5R209	3133333.33	3300000.00	N/D	2800000.00	4256666.67	6800000.00	10233333.33	980000.00
Q5R210	2666666.67	2600000.00	2533333.33	2800000.00	4340000.00	6666666.67	9466666.67	1600000.00
Q5RKT7	20600000.00	17333333.33	60000000.00	23466666.67	34666666.67	27266666.67	26333333.33	10000.00
Q5STU3	N/D	980000.00	1533333.33	1500000.00	3933333.33	2000000.00	3633333.33	3300000.00
Q5SYB0	6433333.333	5600000	5233333.333	5000000	5233333.333	5834666.667	4133333.333	5233333.333
Q5SYQ7	2600000.00	620000.00	100.00	1300000.00	12666666.67	3400000.00	2833333.33	N/D
Q5SYQ8	2600000.00	620000.00	100.00	1300000.00	12666666.67	3400000.00	2833333.33	N/D
Q5SYQ9	2600000.00	620000.00	100.00	1300000.00	12666666.67	3400000.00	2833333.33	N/D
Q5SZC3	N/D	2800000.00	N/D	N/D	5400000.00	8000000.00	7566666.67	800.00
Q5SZC5	2600000.00	100.00	N/D	6666666.67	9900000.00	2200000.00	4733333.33	N/D
Q5T3N0	N/D	1633333.33	1200000.00	1506666.67	2066666.67	3300000.00	723333.33	12000000.00
Q5T3N1	N/D	1633333.33	1200000.00	1533333.33	2500000.00	3300000.00	723333.33	13000000.00
Q5T6W2	N/D	880000.00	2300000.00	2000000.00	300000.00	4800000.00	N/D	9600000.00

Q5TA01	N/D	1466666.67	N/D	N/D	6666666.67	6000000.00	233.33	8300000.00
Q5TA02	N/D	1433333.33	N/D	1400000.00	1520000.00	4666666.67	1900000.00	12000000.00
Q5TCI8	N/D	2433333.33	N/D	2766666.67	8600000.00	9200000.00	2400000.00	15000000.00
Q5TEC6	2266666.67	8400000.00	4000200.00	2000000.00	16666666.67	4866666.67	8400000.00	46666666.67
Q5U077	2433333.33	8600000.00	8700000.00	9000000.00	5233333.33	834666.67	4133333.33	44000000.00
Q5U0A0	N/D	1526666.67	1600000.00	966733.33	866666.67	4200000.00	933333.33	7333333.33
Q5U0I6	N/D	3400000.00	3533333.33	N/D	N/D	300.00	233.33	N/D
Q5UGI3	20600000.00	17333333.33	60000000.00	23466666.67	34666666.67	27266666.67	26333333.33	10000.00
Q5XPV6	N/D	2300000.00	7200000.00	1900000.00	2933333.33	3000000.00	233.33	6866666.67
Q5ZEY3	24000000.00	16400233.33	10233333.33	33333333.33	65933333.33	101333333.33	29433333.33	62000000.00
Q66K91	N/D	2300000.00	7200000.00	1900000.00	2933333.33	3000000.00	233.33	6866666.67
Q6B823	4666666.67	22200000.00	46000333.33	35666666.67	18533333.33	50433333.33	22233333.33	220000000.00
Q6FGE5	N/D	4300000.00	N/D	3000000.00	7166666.67	6800000.00	4333333.33	N/D
Q6FGL0	1866666.67	1730000.00	9300000.00	4633333.33	12666666.67	400666.67	1216666.67	50000000.00
Q6FHK8	N/D	3766666.67	2800000.00	3273333.33	3466666.67	9400000.00	1566666.67	26000000.00
Q6FHU2	N/D	3766666.67	2800000.00	3273333.33	3633333.33	9400000.00	1566666.67	26000000.00
Q6FHZ0	2233333.33	7400000.00	17000000.00	8200000.00	5066666.67	24500000.00	3900000.00	19430000.00
Q6FI37	1333466.67	2800000.00	7400000.00	2600000.00	474333.33	1000666.67	12666666.67	4200000.00
Q6FIA4	1333466.67	2700000.00	7400000.00	2200000.00	10033333.33	6866666.67	11333333.33	100.00
Q6I9V5	N/D	833466.67	933366.67	1966800.00	3600000.00	14000000.00	3833333.33	7333333.33
Q6IBN1	N/D	880000.00	2300000.00	2000000.00	N/D	4666666.67	N/D	300000.00
Q6IC76	N/D	N/D	N/D	N/D	500.00	N/D	3333333.33	N/D
Q6IPF2	N/D	2300100.00	1800000.00	2033400.00	3266666.67	6466666.67	966733.33	24000000.00
Q6LBS1	N/D	2300000.00	7200000.00	1900000.00	2933333.33	3000000.00	233.33	6866666.67
Q6LE88	N/D	1600033.33	12000000.00	1500000.00	4033333.33	3800000.00	2033333.33	N/D
Q6LER6	N/D	2800000.00	6133333.33	1766866.67	90.00	1000.00	3833333.33	12000000.00
Q6NVC0	N/D	833466.67	1133366.67	3033333.33	3666666.67	14000000.00	3833333.33	9400000.00
Q6NVH9	N/D	1496666.67	6233333.33	3266666.67	8000000.00	9466666.67	1083333.33	46666666.67
Q6PK50	3766666.67	2900000.00	467033.33	2233333.33	13000000.00	6633333.33	5033333.33	15000000.00
Q6PK56	1866666.67	400133.33	N/D	6400000.00	8766666.67	5866666.67	9066666.67	N/D
Q6PKA6	2233333.33	566666.67	N/D	2000000.00	980000.00	9166666.67	19133333.33	N/D
Q6PKD2	N/D	6366666.67	200.00	3200000.00	3366666.67	16000000.00	2430000.00	18000000.00
Q6PKH8	N/D	970000.00	N/D	1800000.00	3433333.33	3200000.00	970000.00	3200000.00
Q6PYX1	2600000.00	2300000.00	N/D	4400000.00	12666666.67	4200000.00	5066666.67	N/D
Q6S4P3	2400000.00	1400000.00	N/D	2300000.00	1600000.00	3300000.00	3766666.67	N/D
Q6S8J3	4666666.67	2633333.33	5866666.67	5200333.33	16000233.33	19100000.00	11133333.33	12000000.00
Q6TXQ4	166.67	10166666.67	10600000.00	6333333.33	600.00	800.00	1633433.33	94000000.00
Q6ZNW5	N/D	N/D	N/D	N/D	N/D	2000000.00	4466666.67	6366666.67
Q71DI3	166.67	10166666.67	10600000.00	6333333.33	600.00	800.00	1633433.33	94000000.00
Q71V99	N/D	8100000.00	2000.00	1533333.33	4833333.33	22000000.00	3900000.00	94000000.00
Q75MT9	2233333.33	7400000.00	17000000.00	8200000.00	5066666.67	24500000.00	3900000.00	19430000.00
Q76LA1	N/D	46.67	N/D	100.00	1500000.00	2200000.00	2133333.33	666.67
Q7KYK3	N/D	980000.00	1533333.33	1500000.00	2633333.33	2200000.00	3233333.33	1900000.00
Q7Z474	N/D	3466666.67	4600000.00	553500.00	8900000.00	N/D	2033333.33	18000000.00
Q7Z4Q5	2333333.33	3466666.67	N/D	N/D	1000.00	8766666.67	3733333.33	6966666.67
Q7Z5A3	3533333.33	3300033.33	4300000.00	200.00	3200000.00	6400000.00	2500000.00	4600000.00
Q7Z7J6	2900000.00	2400000.00	2700000.00	4666666.67	5000.00	968000.00	18333333.33	5900000.00
Q7Z7M4	N/D	4200000.00	10166666.67	7100000.00	6933333.33	19000000.00	5300000.00	28000000.00
Q7Z7M5	N/D	3066666.67	10466666.67	4000366.67	4566666.67	9466666.67	4500000.00	29000000.00
Q7Z7M6	N/D	4100000.00	10400000.00	8133333.33	6933333.33	14666666.67	5100000.00	28000000.00
Q7Z7M7	N/D	4100000.00	10400000.00	8133333.33	6933333.33	14666666.67	5100000.00	28000000.00
Q86TY5	1866666.67	1730000.00	9300000.00	4633333.33	12666666.67	400666.67	1216666.67	50000000.00
Q86U12	3933333.33	2800000.00	5866666.67	1900000.00	26666666.67	6000000.00	7033333.33	18000000.00
Q86VG2	N/D	N/D	N/D	1666666.67	N/D	300.00	4366666.67	4333333.33
Q8L7U4	1526666.67	1666666.67	1600000.00	1700000.00	1666666.67	1600000.00	1800000.00	1666666.67
Q8NB78	2500000.00	2459000.00	1970000.00	1466666.67	1559800.00	1968000.00	1833333.33	1590000.00
Q8NE09	2900000.00	2400000.00	2700000.00	2666666.67	2700000.00	2968000.00	1333333.33	2700000.00
Q96C32	20600000.00	17333333.33	60000000.00	23466666.67	34666666.67	27266666.67	26333333.33	10000.00
Q96C96	1526666.67	2166666.67	2800000.00	1996666.67	9256666.67	867133.33	8033333.33	1500000.00
Q96DG6	68000000.00	38666666.67	64333333.33	126670000.00	52666666.67	40002000.00	34333333.33	N/D
Q96FU6	3533333.33	4066666.67	11500000.00	7400000.00	23186666.67	16666666.67	3800666.67	23000000.00
Q96GW1	N/D	800000.00	6133333.33	2666666.67	1067000.00	3800000.00	9600000.00	3800000.00
Q96H31	20600000.00	17333333.33	60000000.00	23466666.67	32666666.67	27266666.67	26333333.33	10000.00
Q96H53	3266666.67	8200000.00	N/D	1500000.00	N/D	3100000.00	6446666.67	N/D
Q96HE7	N/D	1400000.00	N/D	300.00	256666.67	7200000.00	6400000.00	1200000.00
Q96HX7	3933333.33	2800000.00	5866666.67	1900000.00	26666666.67	6000000.00	6366666.67	18000000.00
Q96IS6	7433333.33	7800000.00	17966666.67	5000000.00	15386666.67	10966666.67	11333633.33	8300000.00
Q96KP4	N/D	3300000.00	3400000.00	4200000.00	5500000.00	1700000.00	3566666.67	N/D
Q96QB7	N/D	2533433.33	5433333.33	2566666.67	4166666.67	8800000.00	3400000.00	20000000.00
Q96RE1	N/D	N/D	3700000.00	N/D	13666666.67	4700000.00	3400000.00	1000.00
Q96RS2	N/D	1400000.00	800.00	1300000.00	13000000.00	6466666.67	15666666.67	600.00
Q99729	N/D	N/D	1533333.33	N/D	N/D	4966666.67	7900000.00	9666666.67
Q99757	2066666.67	N/D	N/D	N/D	N/D	2366666.67	6400000.00	3966666.67
Q99933	7433333.33	7800000.00	17966666.67	7433333.33	15386666.67	16966666.67	19333633.33	15300000.00
Q9BRA2	2200000.00	N/D	N/D	N/D	N/D	2200000.00	4366666.67	5800000.00
Q9HAP0	N/D	N/D	N/D	N/D	8900000.00	N/D	4400000.00	N/D
Q9HBB3	N/D	1466666.67	N/D	2200000.00	N/D	4966666.67	1933333.33	8600000.00
Q9NQ81	2800000.00	1400000.00	N/D	630000.00	9453333.33	4000000.00	17333333.33	2600000.00
Q9NQ82	2333333.33	1200000.00	5733333.33	1800000.00	29666666.67	867333.33	22333333.33	6000000.00
Q9NX01	3266666.67	1433333.33	N/D	2000100.00	3466666.67	4000000.00	1746700.00	N/D
Q9NYI7	20000000.00	1500666.67	3300000.00	6133333.33	29333333.33	19000000.00	33066666.67	14000000.00
Q9NZ23	5333333.33	5933333.33	3700000.00	11533333.33	8653333.33	2433333.33	4267000.00	4200000.00
Q9NZE6	2100000.00	980000.00	1600000.00	N/D	3520000.00	3700000.00	6866666.67	5300000.00
Q9P2W1	1600133.33	1600500.00	6313333.33	1906666.67	1906666.67	1750000.00	2700000.00	2150000.00
Q9UG59	N/D	1600033.33	12000000.00	1500000.00	4033333.33	3800000.00	2033333.33	N/D
Q9UIS4	N/D	2300000.00	7200000.00	1900000.00	2933333.33	3000000.00	233.33	6866666.67

Q9UK31	8200000.00	1100000.00	2800000.00	2000.00	41666666.67	16000000.00	20200000.00	23000000.00
Q9UKK4	20666666.67	1300666.67	3200000.00	7466666.67	43333333.33	23333333.33	44033333.33	16666666.67
Q9UN47	20000000.00	1500666.67	3300000.00	6133333.33	32666666.67	26000000.00	47066666.67	14000000.00
Q9UNM1	N/D	7800000.00	6400000.00	10933333.33	5566666.67	9800000.00	1666666.67	62000000.00
Q9UNN8	N/D	N/D	N/D	N/D	3000000.00	2900000.00	700.00	1600000.00
Q9UP60	N/D	N/D	N/D	N/D	1673333.33	1700000.00	2733333.33	N/D
Q9UPN1	N/D	1666666.67	1800000.00	833533.33	1086666.67	3600000.00	2133333.33	N/D
Q9UQC1	1600133.33	3300000.00	N/D	1306666.67	6313333.33	4000100.00	9700000.00	N/D
R4GN08	N/D	1700000.00	2600000.00	1666666.67	726666.67	2900000.00	933333.33	6500000.00
R4GN98	N/D	3800000.00	2300000.00	N/D	1600000.00	3000000.00	6900000.00	1800000.00
R4SBI6	706666.67	N/D	N/D	N/D	680000.00	3120000.00	6700000.00	N/D
S4R3N1	N/D	8800000.00	N/D	10466666.67	2133333.33	700.00	8800000.00	6666.67
S4R3Z2	N/D	2466666.67	N/D	400.00	1853333.33	4300000.00	4600000.00	20000000.00
S6C4S2	N/D	1466666.67	1533333.33	N/D	3266666.67	1800000.00	1833333.33	2500000.00
S6C4S4	N/D	N/D	N/D	N/D	6366666.67	2000000.00	4133333.33	N/D
U3KPS5	N/D	2600000.00	N/D	N/D	3500000.00	N/D	400.00	6400000.00
U3KPZ0	N/D	2166666.67	2400000.00	2433333.33	266.67	9400000.00	3733333.33	12000000.00
U3KQ84	N/D	N/D	N/D	N/D	2966666.67	2000000.00	3333333.33	N/D
U3KQF3	N/D	2166666.67	2400000.00	2433333.33	266.67	9400000.00	3733333.33	12000000.00
U3KQK0	N/D	4400000.00	N/D	5600000.00	3366666.67	2466666.67	4400000.00	16000000.00
U3PXP0	N/D	N/D	1300000.00	N/D	N/D	N/D	N/D	N/D
U6A216	N/D	2600000.00	1733400.00	4000000.00	N/D	N/D	N/D	N/D
V9GYG0	N/D	833466.67	933366.67	1966800.00	3800000.00	13000000.00	3833333.33	7333333.33
V9GZ37	2300000.00	4200000.00	6233333.33	5800000.00	8290000.00	5266666.67	10900000.00	N/D
V9GZ54	N/D	N/D	886666.67	4200000.00	6866666.67	6900000.00	1333.33	N/D
V9HVX6	266.67	900000.00	100.00	1400000.00	16000000.00	3533433.33	4466700.00	240000.00
V9HVZ7	3533333.33	3533333.33	11500000.00	7400000.00	23186666.67	16666666.67	3800666.67	23000000.00
V9HW04	N/D	866733.33	1800000.00	833533.33	1086666.67	3600000.00	2133333.33	N/D
V9HW12	N/D	2016666.67	893333.33	4400000.00	1143333.33	8000000.00	1566666.67	13000000.00
V9HW22	7433333.33	7800000.00	17966666.67	5000000.00	15386666.67	10966666.67	11333633.33	8300000.00
V9HW26	N/D	653466.67	4466666.67	4800000.00	5366666.67	8200000.00	6966666.67	4000000.00
V9HW31	2566666.67	4166666.67	5733333.33	5900000.00	32666666.67	6466666.67	7133466.67	18000000.00
V9HW33	N/D	N/D	6800000.00	100.00	6966666.67	6800000.00	400.00	N/D
V9HW63	460000.00	2366666.67	2300000.00	3533333.33	6933333.33	19000000.00	1460000.00	18000000.00
V9HW65	3333333.33	3233333.33	3600000.00	4266666.67	2000000.00	14666666.67	5100000.00	3000.00
V9HW68	2600000.00	2300000.00	N/D	4400000.00	12666666.67	4200000.00	5066666.67	N/D
V9HW80	2366666.67	980000.00	200.00	N/D	4220000.00	2566666.67	600.00	4600000.00
V9HW84	1866666.67	1800000.00	N/D	2500000.00	10600000.00	400300.00	10963333.33	3200000.00
V9HW85	2000266.67	800.00	2533333.33	7333333.33	18233333.33	11333600.00	27933333.33	28000000.00
V9HWB8	22000000.00	7633333.33	4700000.00	10666666.67	51533433.33	39333333.33	61966666.67	33000000.00
V9HWC6	N/D	4000133.33	1434000.00	2300200.00	8366666.67	24000000.00	566.67	16666666.67
V9HWC7	N/D	2333333.33	N/D	4100000.00	3433333.33	1000.00	3566666.67	19000000.00
V9HWE0	466800.00	1666800.00	3366666.67	3166666.67	4966666.67	8433333.33	867333.33	26000000.00
V9HWE1	3933333.33	2400000.00	3000000.00	706666.67	7466666.67	N/D	2066833.33	N/D
X6R6Z1	N/D	N/D	N/D	300.00	5266666.67	4900000.00	4000000.00	N/D
